# Allosteric coupling from G protein to the agonist binding pocket in GPCRs

**DOI:** 10.1038/nature18324

**Published:** 2016-06-29

**Authors:** Brian T. DeVree, Jacob P. Mahoney, Gisselle A. Vélez-Ruiz, Soren G.F. Rasmussen, Adam J. Kuszak, Elin Edwald, Juan-Jose Fung, Aashish Manglik, Matthieu Masureel, Yang Du, Rachel A Matt, Els Pardon, Jan Steyaert, Brian K. Kobilka, Roger K. Sunahara

**Affiliations:** 1 Department of Pharmacology, University of Michigan Medical School, Ann Arbor, MI 48109; 2 Department of Pharmacology, University of California San Diego School of Medicine, 9500 Gilman Drive, La Jolla, CA 92093; 3 Department of Cellular and Molecular Physiology, Stanford University, Palo Alto, CA; 4 Structural Biology Research Center, VIB, Pleinlaan 2, 1050 Brussels, Belgium; 5 Structural Biology Brussels, Vrije Universiteit Brussel (VUB), Pleinlaan 2, 1050 Brussels, Belgium

**Keywords:** GPCR, nucleotide-free, receptor conformation

## Abstract

G protein-coupled receptors (GPCRs) remain the primary conduit by which cells detect environmental stimuli and communicate with each other^1^. Upon activation by extracellular agonists, these seven transmembrane domain (7TM)-containing receptors interact with heterotrimeric G proteins to regulate downstream second messenger and/or protein kinase cascades^1^. Crystallographic evidence from a prototypic GPCR, the β_2_-adrenergic receptor (β_2_AR), in complex with its cognate G protein, Gs, has provided a model for how agonist binding promotes conformational changes that propagate through the GPCR and into the nucleotide binding pocket of the G protein α-subunit to catalyze GDP release, the key step required for GTP binding and activation of G proteins^2^. The structure also offers hints on how G protein binding may, in turn, allosterically influence ligand binding. Here we provide functional evidence that G protein coupling to β_2_AR stabilizes a ‘closed’ receptor conformation characterized by restricted access to and egress from the hormone binding site. Surprisingly, the effects of G protein on the hormone binding site can be observed in the absence of a bound agonist, where G protein coupling driven by basal receptor activity impedes the association of agonists, partial agonists, antagonists and inverse agonists. The ability of bound ligands to dissociate from the receptor is also hindered, providing a structural explanation for the G protein-mediated enhancement of agonist affinity, which has been observed for many GPCR-G protein pairs. Our studies also suggest that in contrast to agonist binding alone, coupling of a G protein in the absence of an agonist stabilizes large structural changes in a GPCR. The effects of nucleotide-free G protein on ligand binding kinetics are shared by other members of the superfamily of GPCRs, suggesting that a common mechanism may underlie G protein-mediated enhancement of agonist affinity.

Sequencing of the human genome revealed the magnitude of the GPCR superfamily, identifying over 800 genes encoding GPCRs, making this class of receptors the third-largest gene family^3^. Despite the varying nature of the chemical stimuli, which range from photons to small-molecule odorants and hormones to larger peptides and proteins, the generation of G protein-mediated signals proceeds by a common mechanism. Following activation, the receptor engages a heterotrimeric G protein and catalyzes release of GDP from the G protein α-subunit (Gα). Intracellular GTP then binds the nucleotide-free G protein, allowing it to regulate downstream effectors (adenylyl cyclase, phospholipase C, ion channels, etc.) to elicit cellular responses^4^. We recently used x-ray crystallography^2^, hydrogen-deuterium exchange mass spectrometry^5^, and electron microscopy^6^ to characterize an agonist-GPCR-G protein ternary complex in the absence of nucleotide. These studies revealed dramatic conformational changes in G that are stabilized by binding to agonist-activated receptor and provided insight into the mechanism by which GPCRs bind G proteins to promote nucleotide exchange. Here, we suggest an explanation for the allosteric communication which links the nucleotide binding site on the G protein to the hormone binding site on the receptor, with a focus on conformational changes in the extracellular face of the receptor that alter access to the hormone binding site.

GPCR-G protein interactions have historically been monitored using radioligand binding assays. Observations as early as the 1970s suggested that G protein coupling enhances agonist affinity for the receptor, and can be abolished by uncoupling the G protein from the receptor with guanine nucleotides^7^. These and other data formed the basis for the ternary complex model of agonist-receptor-G protein interactions^8,9^. In this paradigm, the active state of the receptor is stabilized by both the agonist and G protein, and enhancement of agonist affinity arises due to the positive cooperativity between agonist and G protein. However, using purified β_2_AR•Gs complexes, we observed peculiar binding characteristics of the antagonist [^3^H]dihydroalprenolol ([^3^H]DHAP) to β_2_AR (Fig. 1a). As illustrated, addition of GDP increases the observed binding of a saturating concentration of [^3^H]DHAP, whereas removal of GDP using a nucleotide lyase, apyrase, decreases [^3^H]DHAP binding. The apyrase-mediated decrease in [^3^H]DHAP binding is reversed upon addition of excess GDP, suggesting that the decrease is indeed due to the formation of nucleotide-free β_2_AR•Gs complexes. Removal of GDP from the β_2_AR•Gs complex relies on the constitutive activity of β_2_AR and the rapid hydrolysis (by apyrase) of GDP released from the α-subunit of Gs, Gsα. The nucleotide-free status of Gsα in these β_2_AR•Gs complexes was confirmed by rapid [^35^S]GTPγS binding kinetics (Extended Data Figure 1)^10^. The observed deficit in [^3^H]DHAP binding to nucleotide-free β_2_AR•Gs is the result of slower [^3^H]DHAP association (Fig. 1b, Extended Data Figure 2). GDP enhances [^3^H]DHAP association in a concentration-dependent manner, with similar effects achieved by complete β_2_AR•Gs uncoupling with GTPγS. Although nucleotides do not significantly affect the affinity (K_d_) of [^3^H]DHAP, their modulatory capacity is gamma phosphate-dependent since GTPγS is ∼10-fold more potent than GDP (Extended Data Figure 3). Thus, β_2_AR bound to nucleotide-free G protein adopts a conformation characterized by restricted access to the hormone binding site.

Crystallographic and pharmacological evidence suggests that the active conformation of β_2_AR is stabilized by nucleotide-free Gs or by a single-chain camelid antibody raised against agonist-bound β_2_AR (nanobody Nb80) (Fig. 2a)^2,11,12^. As illustrated in Fig. 2b (and Extended Data Figure 4a), Nb80 stabilizes a conformation of β_2_AR that restricts [^3^H]DHAP association, similar to nucleotide-free Gs. Importantly, Nb80 also slows the association of full agonist, [^3^H]formoterol (Fig. 2c), as well aspartial agonist, [^3^H]CGP-12177 (Fig. 2d). These data suggest that in the nucleotide-free Gs- or Nb80-stabilized active state, the β_2_AR adopts a ‘closed’ conformation impairing access to the orthosteric binding site, regardless of the orthosteric ligand's cooperativity with G protein. These data agree with our previous observation that the inverse agonist ICI-118,551 blocks the formation of β_2_AR•Gs complexes, but is unable to disrupt pre-formed complexes^10^. Nb80 also impairs binding of inverse agonist [^3^H]carvedilol to β_2_AR by modestly decreasing the observed association rate (Fig. 2e) but significantly decreasing total binding, suggesting that Nb80 and [^3^H]carvedilol do not simultaneously occupy β_2_AR.

Agonist-promoted G protein engagement and subsequent nucleotide loss would be expected to stabilize the active, closed receptor conformation, thus trapping the agonist in the orthosteric site and enhancing its observed affinity. Indeed, uncoupling G protein from receptor using the GTP analog GppNHp has been shown to accelerate agonist dissociation from β_2_AR^13^. Such agonist-G protein cooperativity is not predicted for neutral antagonists like alprenolol, which do not stimulate G protein coupling and thus should not stabilize the closed conformation. However, we have previously demonstrated that Gs can be ‘forced’ to form a complex with β_2_AR bound to antagonist alprenolol^10^, provided that free nucleotide is removed, indicating that antagonist-bound β_2_AR retains enough basal activity to engage Gs. Consistent with this model, Figures 2f and Extended Data Figure 4b clearly illustrate a progressive slowing of [^3^H]DHAP (or [^3^H]CGP-12177, data not shown) dissociation in response to increasing Nb80 concentrations, suggesting that Nb80-mediated stabilization of the closed, active receptor conformation can trap [^3^H]DHAP in the orthosteric binding site.

Analysis of access to the hormone binding sites in inactive- and active-state β_2_AR structures provides a structural rationale for the slowing of agonist and antagonist association (Figs. 3, Extended Data Figure 6 and Supplementary Video 1). The binding of Gs or Nb80 to β_2_AR stabilizes a rearrangement of the cytoplasmic end of TM7 (Fig. 4a & b) immediately above the ligand binding site and a change in the structure of the extracellular loop between TM4 and TM5 (ECL2). In comparison to the inactive β_2_AR, the structure of the β_2_AR-Gs or -Nb80 (or related Nb6B9^14^) complex identifies two aromatic residues, Phe193^(5.25 or ECL2)^ and Tyr308^7.35^, that move approximately 2-2.5 Å closer to each other to form a lid-like structure over the orthosteric binding site. Lys305^7.32^ also contributes to capping the orthosteric site by trading its salt bridge^15^ with Asp192^ECL2^ for an interaction with the backbone carbonyl of Phe193^ECL2^ (Fig. 4c). These structural changes are stabilized in the active forms of β_2_AR bound to either the ultra-high affinity agonist BI-167107 or the smaller, low affinity agonist adrenaline^15^, and formation of this ‘lid’ would be expected to sterically obstruct both ligand association and dissociation.

To validate this structural model, we tested whether a residue smaller than tyrosine could modify the capacity of Nb80 to slow ligand association. Mutation of Tyr308^7.35^ to alanine, previously shown to lower agonist affinity for β_2_AR^16^, significantly diminishes the capacity of Nb80 to slow the association of [^3^H]DHAP and even the agonist [^3^H]formoterol (Extended Data Figure 5), as suggested by recent molecular dynamics simulations^17^. Interestingly and in contrast to [^3^H]DHAP association, preincubation with 10 μM Nb80 also enhances the *extent* of [^3^H]formoterol binding in the Y308A mutant. Eliminating barriers that impairs access to the orthosteric site (*eg*. Y308A) allows the agonist to at least enter the receptor where it can stabilize nanobody binding. The enhancement, therefore, is a reflection of the capacity of the agonist [^3^H]formoterol to cooperatively stabilize Nb80 binding and vice-versa and concomitantly slow the *dissociation* of the bound agonist (Extended Data Figure 5d). The data also suggest that while Tyr308^7.35^ significantly limits access to the orthosteric site, other residues may work in concert with Tyr308^7.35^ in the active β_2_AR conformation to slow agonist dissociation.

It is noteworthy that the movement of Phe193^ECL2^ and Tyr308^7.35^ is not fully observed in the crystal structure of β_2_AR bound to an agonist alone^18^, nor in the inactive-state structure of β_1_AR bound to the agonist isoprenaline^19^ (Extended Data Figure 6, Supplementary Video 1, and Supplementary Video 2). Binding of G protein or G protein-mimetic (nanobody) is sufficient to stabilize the closed, active conformation since their effects on ligand binding kinetics (as in Figs. 1 and 2) are agonist-independent. An agonist may enhance G protein engagement but poorly stabilizes the closed, active conformation by itself. Additionally, the data presented here suggest that formation of the closed, active conformation stabilized by the nucleotide-free G protein can occur due to basal receptor activity, in keeping with predictions of more recent models of GPCR pharmacology such as the extended and cubic ternary complex models^20,21^ (see supplemental materials for extended discussion). Moreover, conformational changes stabilized by the nucleotide-free G protein influence not only agonist binding, but ligand binding in general, implying that the role of nucleotides needs to be included in an updated version of ternary complex model.

The capacity of G proteins to stabilize a closed receptor conformation explains the poorly defined GTPγS-mediated increase in radiolabeled antagonist binding observed with several GPCRs, including muscarinic, α-adrenergic, adenosine, and opioid receptors^22–25^ (as in Extended Data Figure 7 & Extended Data Figure 8). We analyzed the behavior of the M2 muscarinic acetylcholine receptor (M2R) and the μ-opioid receptor (MOPr) to determine whether GTPγS-mediated uncoupling relieves a G protein-stabilized closed conformation. We focused on these receptors since structural models are available for both inactive and active conformations^26-29^, and to determine whether the mechanism we propose for β_2_AR is shared among other GPCRs. The active-state structure of the M2R, in particular, revealed similar conformational changes to the β_2_AR in that a ‘lid-like’ structure is formed above the orthosteric site^27^ (see Supplementary Video 4 & Supplementary Video 5). Although the structural changes are not identical, the effect of G proteins (or nanobodies) on the association and dissociation of ligands at the orthosteric sites is shared among β_2_AR, M2R, and MOPr (Extended Data Figure 9 and Supplementary Video 3), suggesting that the allosteric effects of G proteins on orthosteric agonists may be manifested by conceptually common mechanisms. More discussion of the details and implications are described in the supplementary materials. Additionally, many recent studies have focused on the allosteric effect of sodium ions on Class A GPCR ligand binding and signaling^30^. Outward movement of TM6 during receptor activation collapses the sodium binding pocket in many Class A GPCRs, thus it appears that loss of bound sodium is necessary for G proteins to stabilize a closed, active receptor conformation.

The formation of the closed conformation also has particular significance to the development of allosteric modulators targeting Class A GPCRs. Most allosteric modulator binding sites have focused on the extracellular vestibule located above the orthosteric binding sites. For the muscarinic M2R for example, the potent allosteric positive modulator LY2119620 utilizes residues that form the ‘lid’ in the active, closed conformation as described here, as the floor of the vestibule^27^. Stabilization of this closed conformation may therefore be an important aspect on the differentiation between positive allosteric modulators, which enhance agonist binding and activation, and negative allosteric modulators, which decrease agonist binding.

In summary, we provide pharmacological and biochemical evidence suggesting that the closed, active conformation of GPCRs is stabilized by the nucleotide-free G protein, allowing G proteins to influence passage of ligands to the orthosteric binding site. The dramatic effect of G proteins on either ligand association or dissociation is consistent with, and in fact validates, structural models generated from x-ray crystallography where G protein coupling on the intracellular face of the receptor allosterically influences the structure of the extracellular face. Agonist or hormone binding enhances G protein engagement, where by formation of the active receptor conformation is accompanied by nucleotide loss from the G protein. Therefore, the capacity of G proteins to enhance agonist binding affinity is structurally and energetically linked to the agonist's capacity to promote nucleotide loss from Gα.

## Methods

### Large-scale Purification of β_2_AR

β_2_AR bearing an N-terminal FLAG tag and C-terminal 10×-His tag was expressed in *Sf*9 cells (Invitrogen) and purified as previously described^2^.

### Expression and purification of G protein and Nanobodies

Gs and Go heterotrimer were expressed in HighFive™ (Invitrogen) insect cells using recombinant baculovirus and purified by chromatography on Ni-NTA, MonoQ, and Superdex 200 resin, as previously described^31^. Nanobodies were expressed in *Escherichia coli* and purified as previously described^11,14,27^.

### Membrane Preparations

HEK293T cells (ATCC) were used for small-scale expression and purification of β_2_AR and mutants. Cells were grown in DMEM + 10% FBS to ∼70% confluency, then transfected with mYFP-β_2_AR (pCMV5, 6 μg DNA per 10-cm plate) using Lipofectamine 2000. Cells were harvested 40-48 hrs post-transfection in ice-cold lysis buffer buffer (50 mM HEPES, pH 8.0, 65 mM NaCl, 1 mM EDTA, 35 μg/ml phenylmethylsulfonyl fluoride, 32 μg/ml each tosyl-L-phenylalanine-chloromethylketone and tosyl-L-lysine-chloromethylketone, 3.2 μg/ml leupeptin, 3.2 μg/ml ovomucoid trypsin inhibitor). The cell suspension was sonicated using a Branson Sonifier and centrifuged for 20 minutes at 25,000*g*. The pellet was resuspended in wash buffer (50 mM HEPES, pH 8.0, 100 mM NaCl with protease inhibitors listed above) using a Dounce homogenizer, then centrifuged for 20 minutes at 25,000*g*. The pellet was resuspended and homogenized in minimal wash buffer and the volume was adjusted to reach a final protein concentration of 5 mg/ml as measured by the Bradford protein assay. Membranes were frozen by slowly pouring into liquid nitrogen and stored at -80 °C until use.

### Enrichment of β_2_AR and β_2_AR-Y308A from HEK293T cells

Frozen membranes were thawed on ice and NaCl, MgCl_2_, and GTPγS were added to reach final concentrations of 300 mM, 1 mM, and 10 μM, respectively. Timolol was then added to a final concentration of 1 μM and the membranes were incubated for 10 minutes on ice. Receptors were solubilized for 1 hour at 4 °C in the presence of 1% dodecylmaltoside (DDM) and 0.1% cholesterol hemisuccinate (CHS). Following centrifugation for 30 minutes at 25,000g, the supernatant was applied to Ni-NTA agarose. The column was slowly washed with 20 column volumes of 20 mM HEPES, pH 8.0, 300 mM NaCl, 0.1% DDM, 0.01% CHS to remove bound timolol. Receptor was eluted in the same buffer plus 200 mM imidazole and concentrated using an Amicon 30 kDa-cutoff spin concentrator for addition to the rHDL reconstitution mixture.

### Receptor Reconstitution into rHDL Particles

Reconstitutions were performed as described^32^, with the amount of receptor added never exceeding 20% of the total reaction volume. For samples that contained Gs, the purified heterotrimer was added to the pre-formed β_2_AR-rHDL particles, incubated for 2 hours at 4°C, and BioBeads (Bio-Rad) were used to remove the added detergent. Nucleotide-free Gs•β_2_AR complex was prepared by incubating β_2_AR-Gs-rHDL particles with apyrase in the presence of 1 mM MgCl_2_ for 30 minutes at room temperature, or alternately, 2 hours at 4°C. If needed, the sample was passed through a Superdex 200 gel filtration column to remove free nucleotide and apyrase.

### Radioligand Association Experiments using rHDL Particles

All assays were performed in Tris-buffered saline (TBS; 25 mM Tris-HCl, pH 7.4, 136 mM NaCl, 2.7 mM KCl) with a final concentration of 0.05% w/v bovine serum albumin. Reaction components were mixed and pre-incubated at room temperature (see below) before the addition of radioligand to initiate the association time course. Aliquots were withdrawn at the indicated times and filtered over Whatman GF/B filters pre-soaked in 0.3% w/v polyethyleneimine. Filters were washed with ice-cold TBS, dried, and subjected to liquid scintillation counting on a TopCount™ NXT (Perkin-Elmer, MA). Bound ligand never exceeded 10% of the total ligand added.

#### Kinetic binding experiments with [^3^H]DHAP and Nb80, β_2_AR-rHDL

For association experiments, receptor in rHDL was pre-incubated with varying concentrations of Nb80 and the reaction was started by addition of 5 nM [^3^H]DHAP (Perkin Elmer). For dissociation experiments, the samples were first incubated with 5 nM [^3^H]DHAP for 30 minutes, followed by incubated with varying Nb80 concentrations for 30 minutes. The reaction was started by adding 50 μM cold alprenolol. Non-specific binding was determined in the presence of 10 μM (+/-)-propranolol.

#### Binding experiments with [^3^H]DHAP and Gs•β_2_AR nucleotide-free complexes

For association experiments, gel-filtered samples of apyrase-treated Gs•β_2_AR-rHDL particles were incubated with 5 nM [^3^H]DHAP to bind any receptor that was not complexed with Gs. The experiment was started by adding varying amounts of either GDP or GTPγS. For “equilibrium” binding experiments, samples were incubated with all the indicated components at room temperature for 90 minutes before filtration. Non-specific binding was determined in the presence of 10 μM (+/-)-propranolol.

#### [^3^H]Formoterol Association to β_2_AR

β_2_AR-rHDL was incubated with the indicated concentrations of Nb80 for 30 minutes at room temperature. [^3^H]formoterol (Perkin Elmer) was added to reach 10 nM final concentration. These assays also contained 1 mM ascorbic acid in the reaction buffer. Non-specific binding was determined in the presence of 10 μM (+/-)-propranolol.

#### [^3^H](-)-CGP-12177 Association to β_2_AR

β_2_AR-rHDL was incubated with the indicated concentrations of Nb80 for 30 minutes at room temperature. [^3^H](-)-CGP-12177 (Perkin Elmer) was added to reach 1 nM final concentration. Non-specific binding was determined in the presence of 10 μM (+/-)-propranolol.

#### [^3^H]Carvedilol Association to β_2_AR

Due to high amounts of non-specific [^3^H]carvedilol (American Radiolabeled Chemicals) binding both to bovine serum albumin (BSA) and to the glass fiber filters typically used for separation, β_2_AR-rHDL was diluted into empty rHDL particles rather than into a 5× BSA solution (0.25% w/v BSA in TBS buffer) prior to addition to the assay mix. Using empty rHDL in place of BSA was critical for maintaining sample recovery from the assay plate while improving the signal-to-noise ratio of the assay. The receptor was incubated with the indicated concentrations of Nb80 for 15 minutes at room temperature, then for 30 minutes at 4°C. [^3^H]carvedilol was added to reach 1 nM final concentration. Aliquots were withdrawn at the indicated time points and bound ligand was isolated using gel filtration on Sephadex G75 resin. Non-specific binding was determined in the presence of 10 μM (+/-)-propranolol.

## Extended Data

**Extended Data Figure 1:**
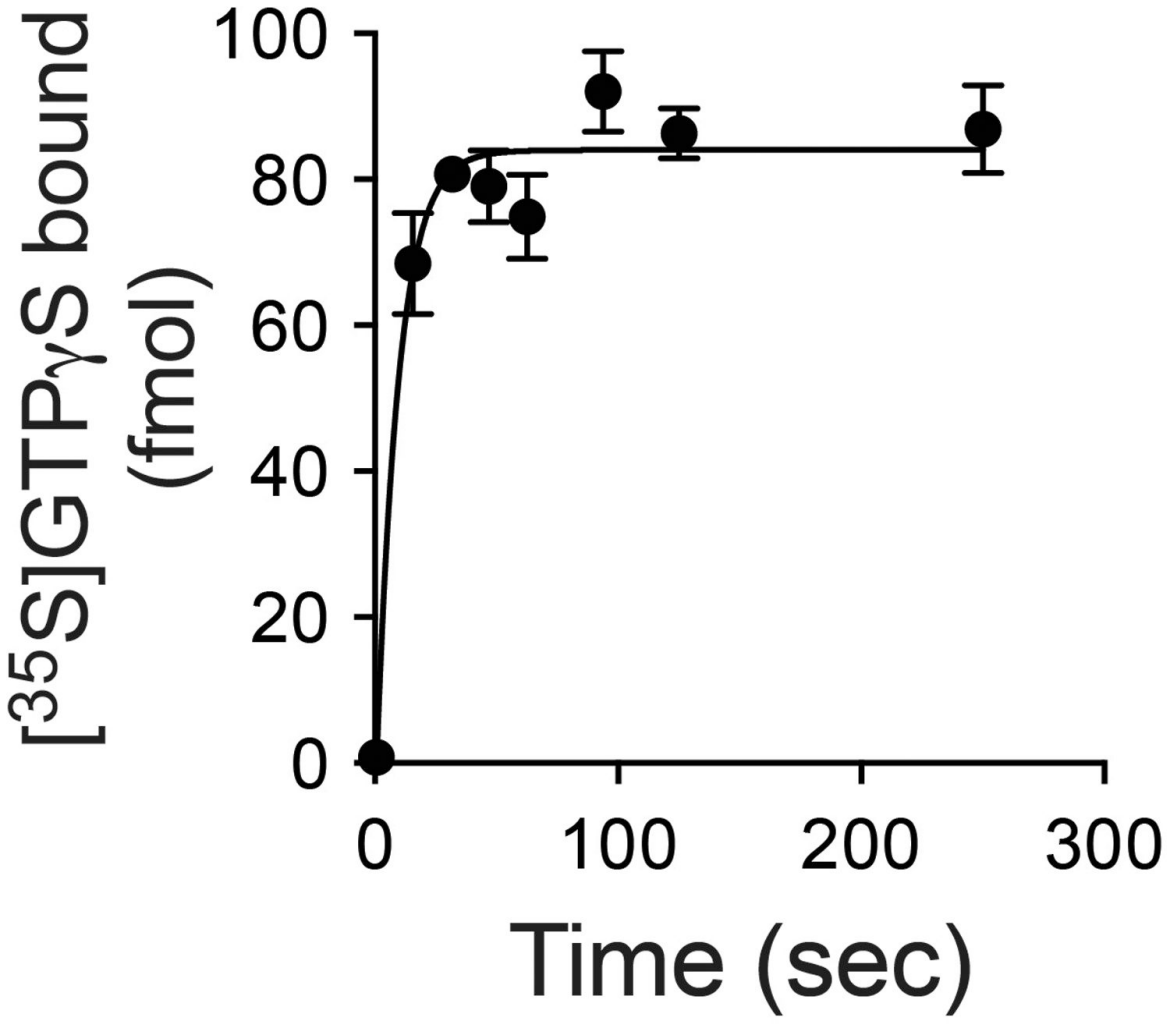
Confirmation of nucleotide removal from β_2_AR•Gs by apyrase Gs and Flag-tagged β_2_AR were reconstituted in rHDL and treated with the non-specific nucleotide lyase, apyrase. Samples were applied to an anti-Flag affinity resin to remove products of the GDP degradation (GMP and P_i_). Samples were incubated with 100 nM [^35^S]GTPγS at room temperature. At various times, samples were subjected to rapid filtration through glass fiber filters (GF/B) followed by 10 volumes of ice-cold buffer washes containing 10 μM GDP. Filters were dried and subjected to liquid scintillation counting (Top-Count™, Perkin-Elmer). To a first approximation the rapid binding event suggests that the complex is empty of nucleotide, based on the limited temporal resolution of this mixing and filtration technique. [^3^H]DHAP and [^35^S]GTPγS binding to the reconstituted complex yields a final R:G ratio of 1:0.95, suggesting that up to 95% of the β_2_AR-rHDL particles contain a single functional G protein. This suggest that only those G proteins associated with the β_2_AR will bind [^35^S]GTPγS within this time frame in the absence of receptor agonists. Data are shown as mean ± SEM from n=3 independent experiments performed in duplicate.

**Extended Data Figure 2:**
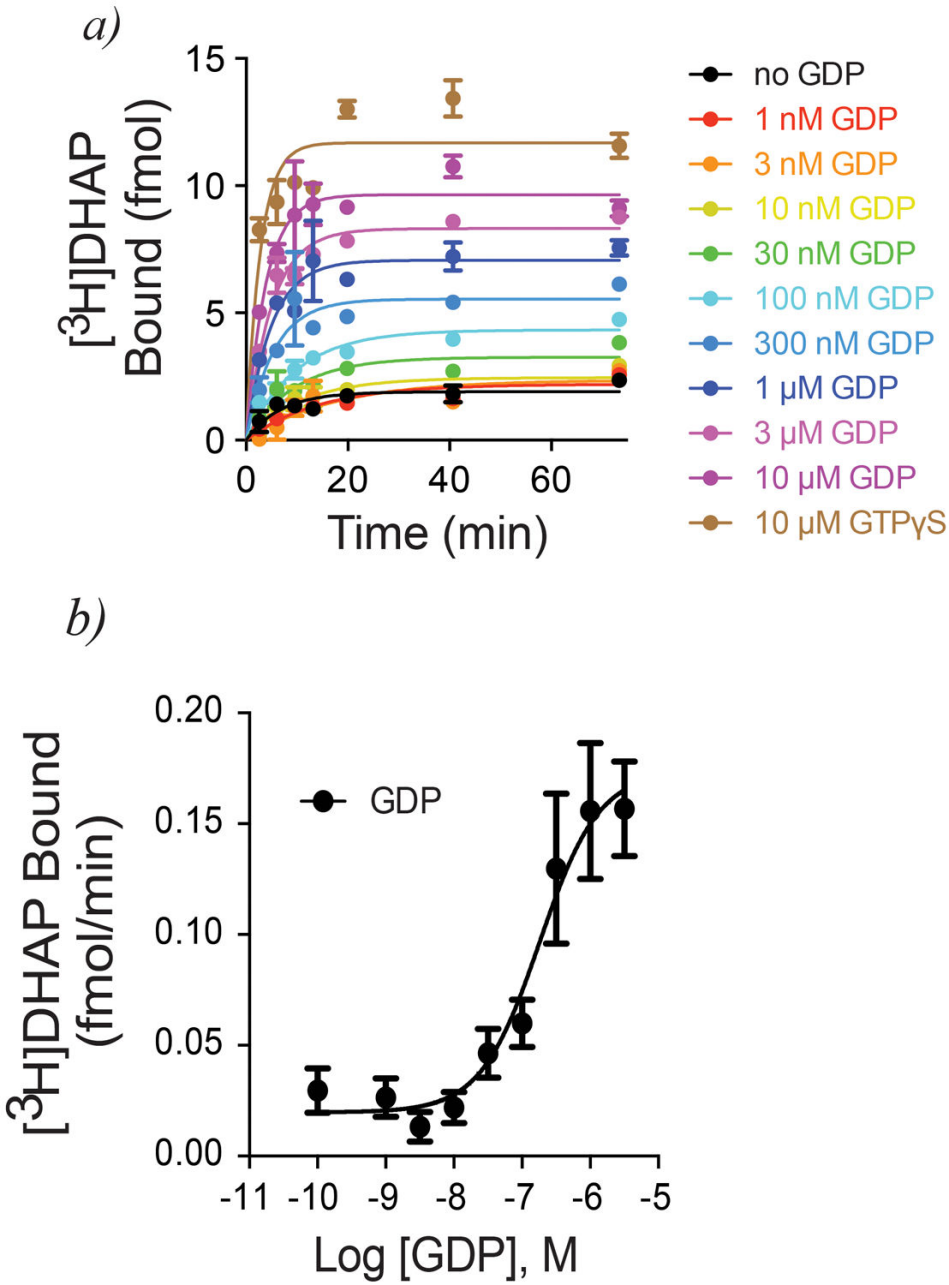
GDP accelerates [^3^H]DHAP binding to β_2_AR•Gs ***a)*** Time course monitoring [^3^H]DHAP association to apyrase-treated β_2_AR•Gs complexes in the presence of varying GDP concentrations. GDP increases both the observed association rate constant and the maximum binding of [^3^H]DHAP. ***b)*** Concentration-response showing enhancement of the observed [^3^H]DHAP association rate constant by GDP (EC_50_ = 181 ± 66 nM). All data are shown as mean ± SEM from n=3 independent experiments performed in duplicate.

**Extended Data Figure 3:**
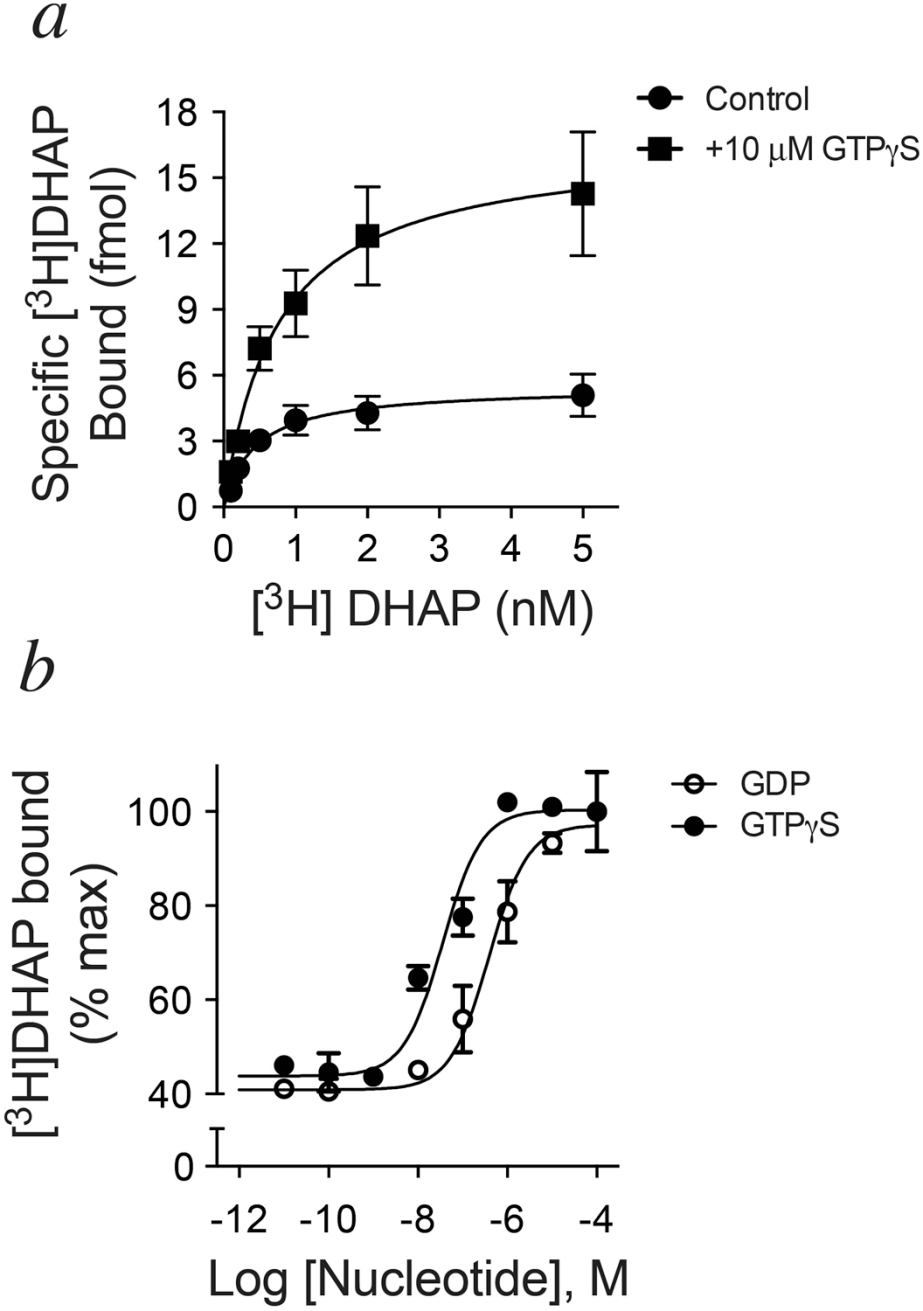
Effect of guanine nucleotides on [^3^H]DHAP binding to β_2_AR•Gs ***a)*** In saturation binding assays, addition of GTPγS to apyrase-treated β_2_AR•Gs complexes increased the observed B_max_ for [^3^H]DHAP without significantly altering K_d_ (Control: B_max_ = 5.5 ± 0.52 fmol, K_d_ = 0.88 nM; +GTPγS: B_max_ = 16.6 ± 1.9 fmol, K_d_ = 0.56 nM) ***b)*** Both GDP and GTPγS could enhance maximal [^3^H]DHAP binding in a concentration-dependent manner (GDP Log(EC_50_) = -6.42 ± 0.12, or EC_50_ ∼386 nM; GTPγS Log(EC_50_) = -7.45 ± -0.16, or EC_50_ ∼35 nM). All data are shown as mean ± SEM from n=3 independent experiments performed in duplicate.

**Extended Data Figure 4:**
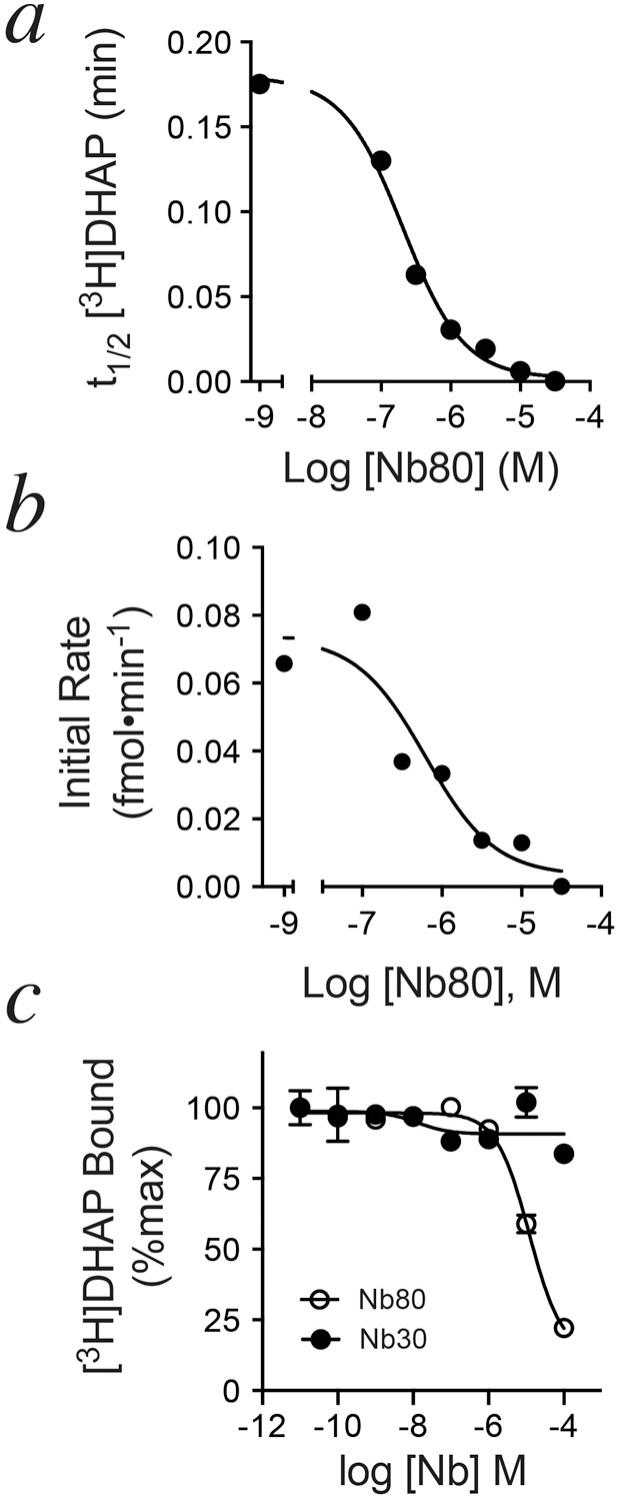
Effect of Nb80 on antagonist binding to β_2_AR ***a)*** Association of [^3^H]DHAP is progressively slowed following pre-incubation of β_2_AR with increasing concentrations of Nb80. ***b)*** If [^3^H]DHAP is allowed to first equilibrate with β_2_AR, Nb80 slows [^3^H]DHAP dissociation from β_2_AR in a concentration-dependent manner. ***c)*** Due to the dramatic slowing of [^3^H]DHAP binding kinetics, Nb80 (but not a control nanobody, Nb30, which has no effect on agonist affinity for β_2_AR) appears competitive with [^3^H]DHAP if insufficient time is given to reach equilibrium. Data shown are from assays incubated 90 minutes at room temperature. All data are shown as mean ± SEM from n=3 independent experiments performed in duplicate.

**Extended Data Figure 5:**
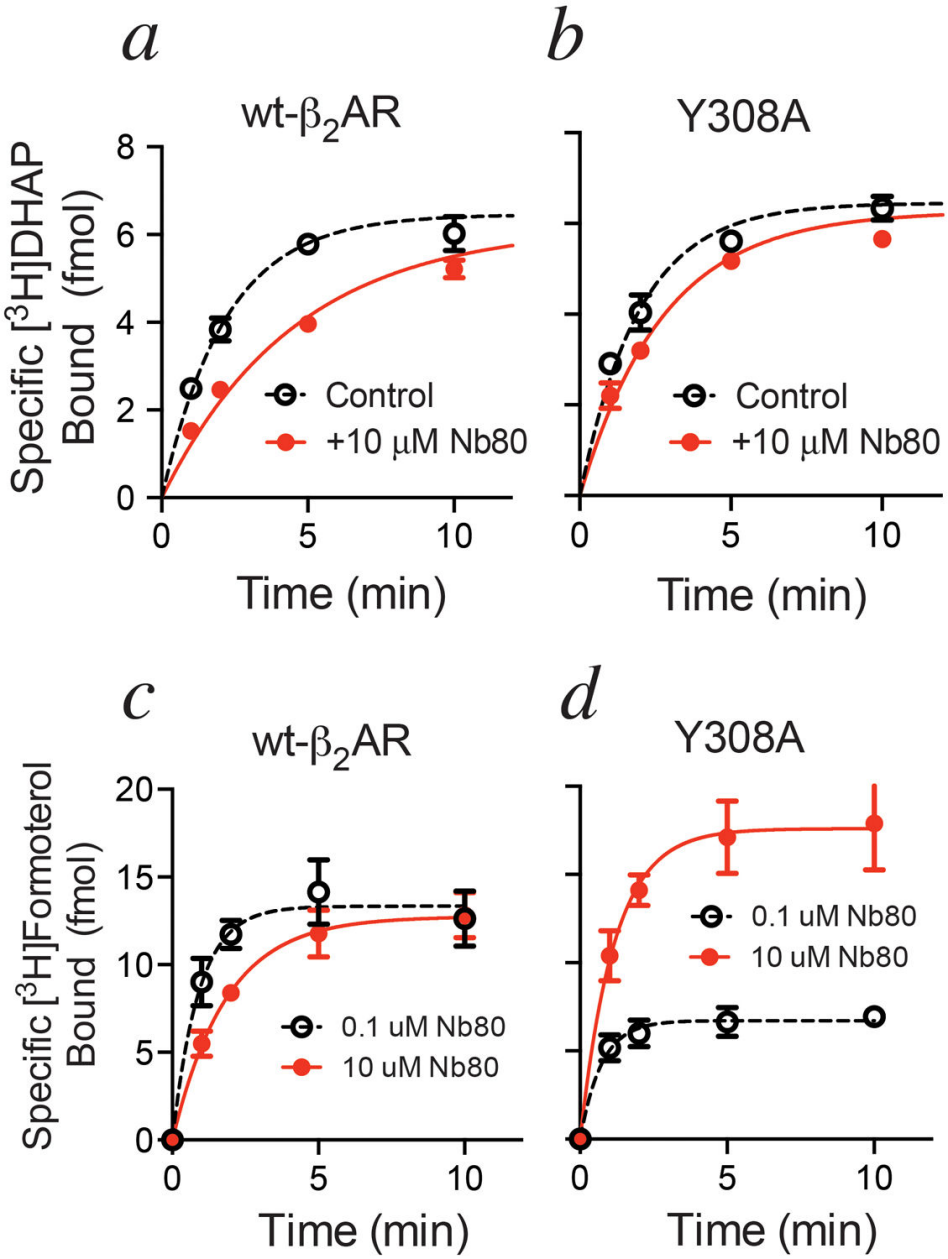
Y308A mutation abolishes the rate-slowing effects of Nb80 ***a) and b)*** Time course of [^3^H]DHAP binding to wild-type β_2_AR **(*a*)** or β_2_AR-Y308A **(*b*)** following pre-incubation of receptor with Nb80. Nb80 significantly slowed [^3^H]DHAP association to wild-type β_2_AR (-Nb80 k_obs_ = 0.45 ± 0.05 min^-1^ or t½ = 1.5 ± 0.2 min, +Nb80 k_obs_ = 0.20 ± 0.03 min^-1^ or t½ = 3.5 ± 0.5 min; p = 0.011 by an unpaired two-tailed *t*-test), but less effectively slowed [^3^H]DHAP association to β_2_AR-Y308A (-Nb80 k_obs_ = 0.50 ± 0.06 min^-1^ or t½ = 1.4 ± 0.2 min; +Nb80 k_obs_ = 0.32 ± 0.01 min^-1^ or t½ = 2.2 ± 0.1 min; p = 0.05 by an unpaired two-tailed t-test. All data are shown as mean ± SEM from n=4 (-Nb80) or n=3 (+Nb80) independent experiments performed in duplicate. ***c)*** and ***d)*** Time course of [^3^H]formoterol binding to wild-type β_2_AR **(*c*)** or β_2_AR-Y308A **(*d*)** following pre-incubation of receptor with Nb80. Nb80 slowed [^3^H]formoterol association to wild-type β_2_AR (0.1 μM Nb80 k_obs_ = 0.68 ± 0.13 min^-1^ or t½ = 1.0 ± 0.2 min, 10 μM Nb80 k_obs_ = 0.27 ± 0.05 min^-1^ or t½ = 2.6 ± 0.5 min; p = 0.031 by an unpaired two-tailed *t*-test). However, with β_2_AR-Y308A, Nb80 had little effect on the observed association rate constant but enhanced the amount of [^3^H]formoterol bound (0.1 μM Nb80 k_obs_ = 0.37 ± 0.11 min^-1^ or t½ = 1.9 ± 0.6 min with a plateau of 10.1 ± 0.8 fmol, 10 μM Nb80 k_obs_ = 0.53 ± 0.13 min^-1^ or t½ = 1.3 ± 0.4 min with a plateau of 21.3 ± 1.2 fmol; unpaired two-tailed *t*-test of the k_obs_ values showed p = 0.4). All data are shown as mean ± SEM from n=4 independent experiments performed in duplicate.

**Extended Data Figure 6:**
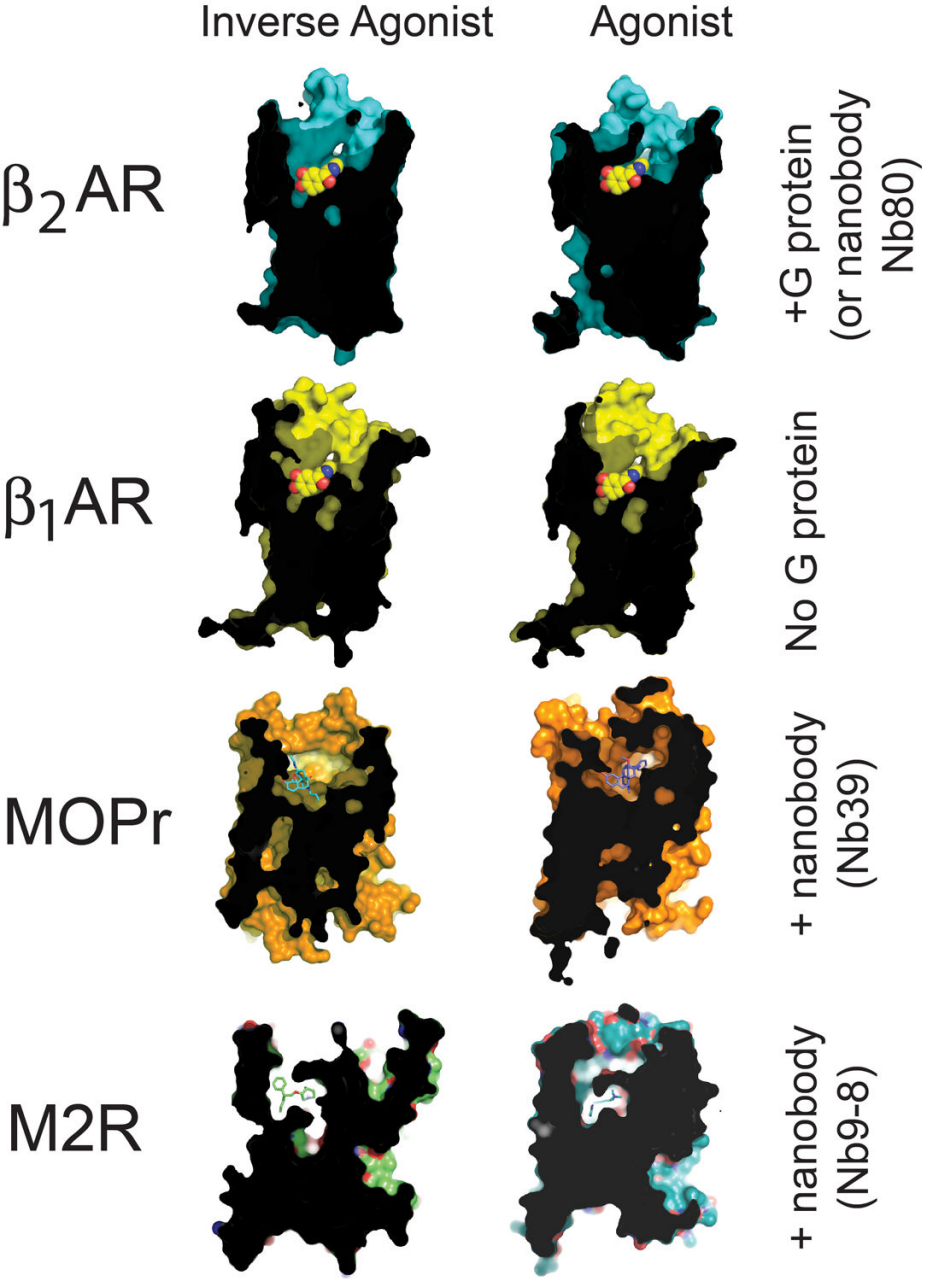
The closed conformation stabilized by agonist and G protein (or mimic) Illustrated are the crystal structures of agonist- vs. inverse agonist-bound of the β_2_AR (cyan) and β_1_AR (yellow), where only β_2_AR is bound to G protein. Similarly, the μ-opioid receptor (MOPr, orange) adopts a closed conformation upon binding G protein surrogate, Nb39. (β_2_AR; PDB 2RH1, β_2_AR•Gs; PDB 3SN6, β_1_AR; PDB 2YCW, β_1_AR-iso; PDB 2Y03, MOPr; PDB 4DKL, MOPr-Nb39; PDB 5C1M, M2R; PDB 3UON, M2R-Nb9-8; PDB 4MQS).

**Extended Data Figure 7:**
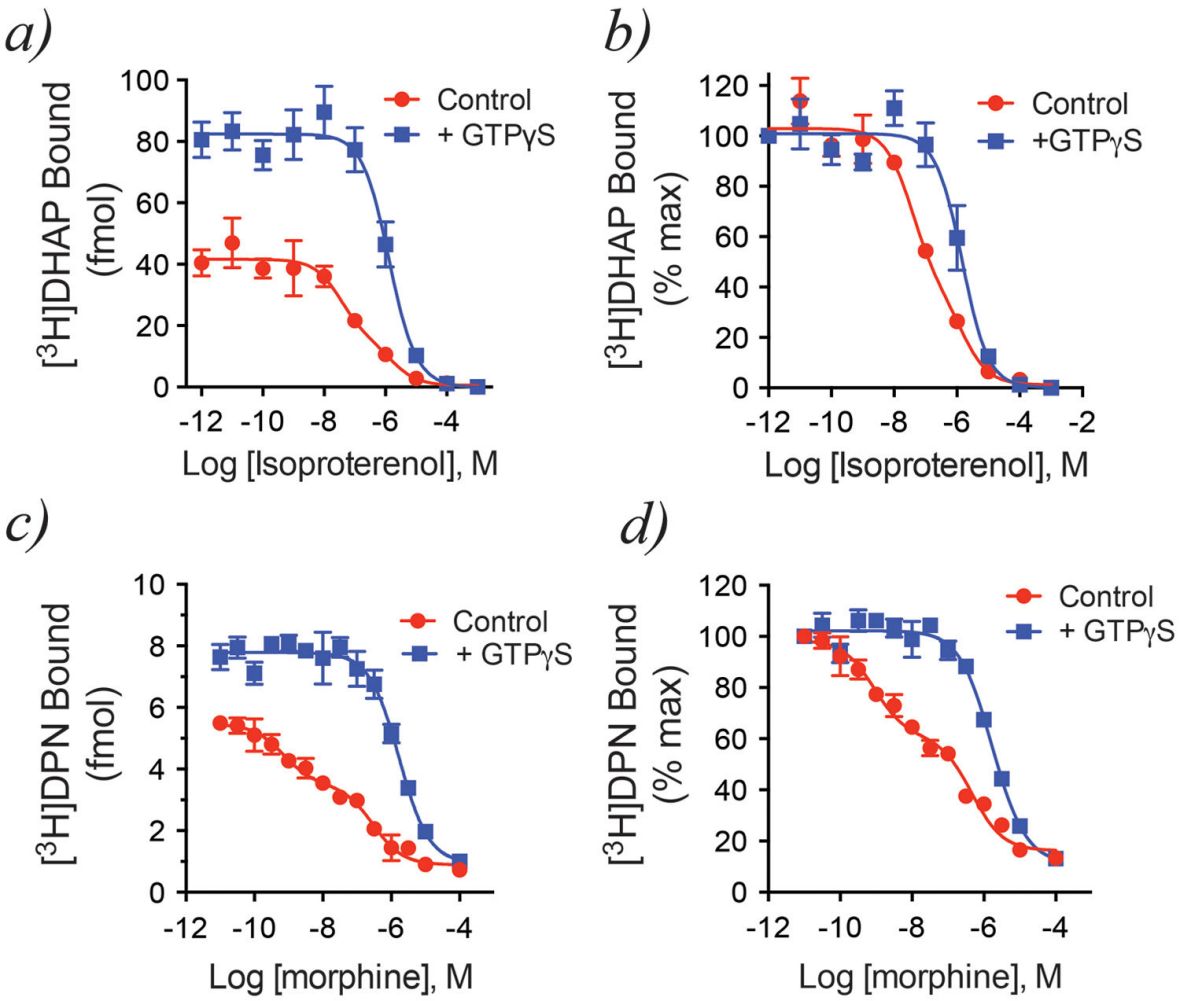
Effect of guanine nucleotides on [^3^H]antagonist binding are also seen in competition binding assays ***a)*** Agonist (isoproterenol) competition binding using apyrase-treated β_2_AR•Gs complexes shows the characteristic G protein-dependent shift in agonist affinity, along with a dramatic increase in total [^3^H]DHAP binding, upon the addition of 10 μM GTPγS. ***b)*** Normalization of the data from ***a)*** yields a plot representative of what is commonly reported in the literature. ***c)*** Similar to β_2_AR, agonist (morphine) competition binding using MOPr•Go complexes shows the characteristic G protein-dependent shift in agonist affinity, along with a dramatic increase in total [^3^H]DPN binding, upon the addition of 10 μM GTPγS. ***d)*** Normalization of the data from ***c)***. All data are shown as mean ± SEM from n=3 independent experiments performed in duplicate.

**Extended Data Figure 8:**
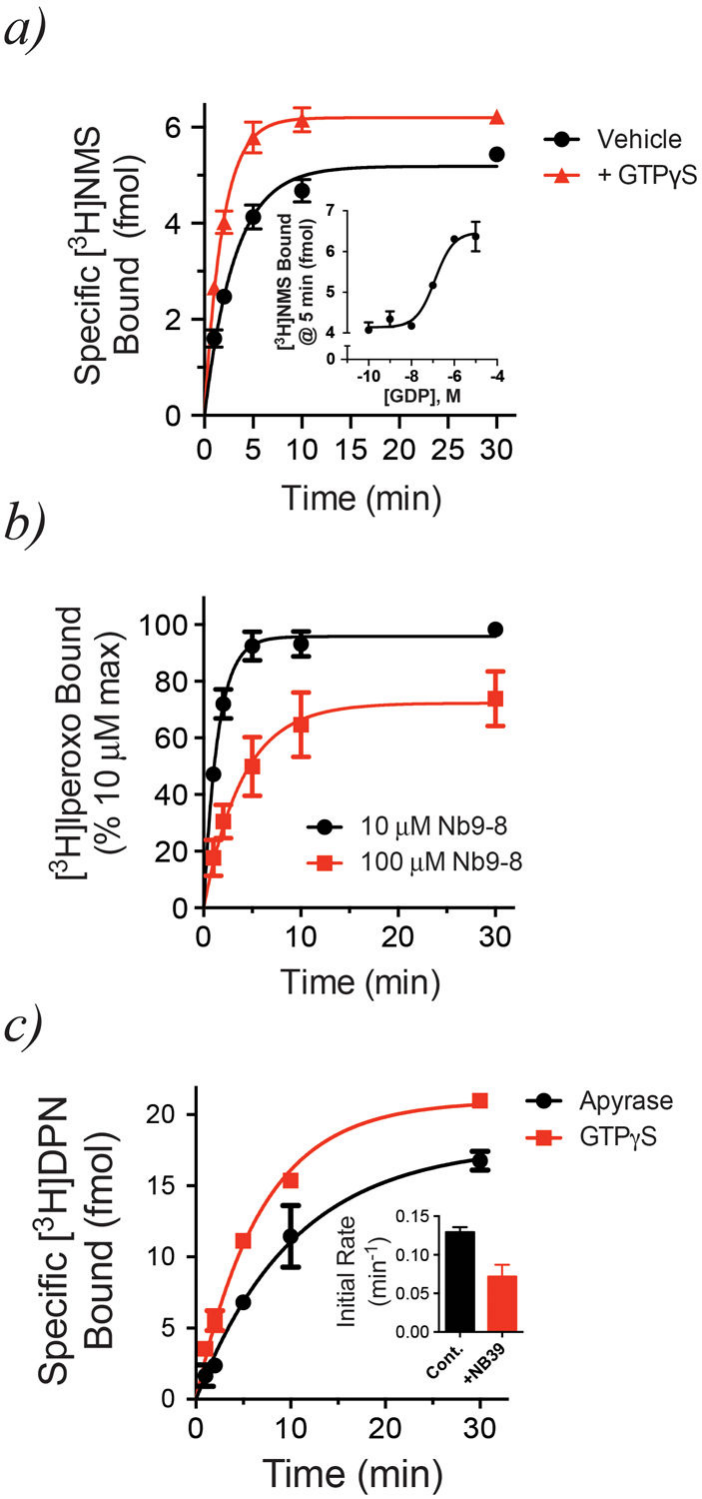
Mu opioid receptor and M2 muscarinic acetylcholine receptor behave similarly to β_2_AR when bound to nucleotide-free G protein or an active-state stabilizing nanobody ***a)*** Following apyrase treatment of M2R•Go complexes, addition of 10 μM GTPγS enhances association of [^3^H]N-methylscopolamine ([^3^H]NMS) to M2R (Vehicle k_obs_ = 0.32 ± 0.02 min^-1^ or t½ = 2.2 ± 0.1 min, +GTPγS k_obs_ = 0.54 ± 0.02 min^-1^ or t½ = 1.3 ± 0.1 min; p = 0.002 by an unpaired two-tailed *t*-test). Data are shown as mean ± SEM from n=3 independent experiments performed in duplicate. Addition of GDP was also able to increase the rate of [^3^H]NMS binding (inset; pEC_50_ = 6.91 ± 0.18 or EC_50_ ∼123 nM; mean ± SEM from n=2 independent experiments performed in duplicate). ***b)*** Pre-treatment of M2R with either 10 μM (black circles) or 100 μM (red squares) Nb9-8^27^ impairs association of [^3^H]iperoxo to M2R (10 μM Nb9-8 k_obs_ = 0.68 ± 0.09 min^-1^ or t½ = 1.0 ± 0.2 min, 100 μM Nb9-8 k_obs_ = 0.25 ± 0.04 min^-1^ or t½ = 2.8 ± 0.5 min; p = 0.04 by an unpaired two-tailed *t*-test). Data are shown as mean ± SEM from n=3 (10 μM Nb9-8) or n=2 (100 μM Nb9-8) independent experiments performed in duplicate. ***c)*** Addition of 10 μM GTPγS to apyrase-treated MOPr•Go complexes hastened association of the antagonist [^3^H]diprenorphine ([^3^H]DPN) to MOPr (Apyrase k_obs_ = 0.06 ± 0.02 min^-1^ or t½ = 9.8 ± 1.3 min, +GTPγS k_obs_ = 0.12 ± 0.01 min^-1^ or t½ = 5.6 ± 0.6 min; p = 0.1 by an unpaired two-tailed *t*-test). The effect of nucleotide-free G protein was recapitulated by pre-incubating MOPr with Nb39^28^ (inset; control k_obs_ = 0.13± 0.01 min^-1^, +100 μM Nb39 k_obs_ = 0.07 ± 0.02 min^-1^). Data are shown as mean ± SEM from n=2 (MOPr•Go) or n=3 (MOPr + Nb39) independent experiments performed in duplicate.

**Extended Data Figure 9:**
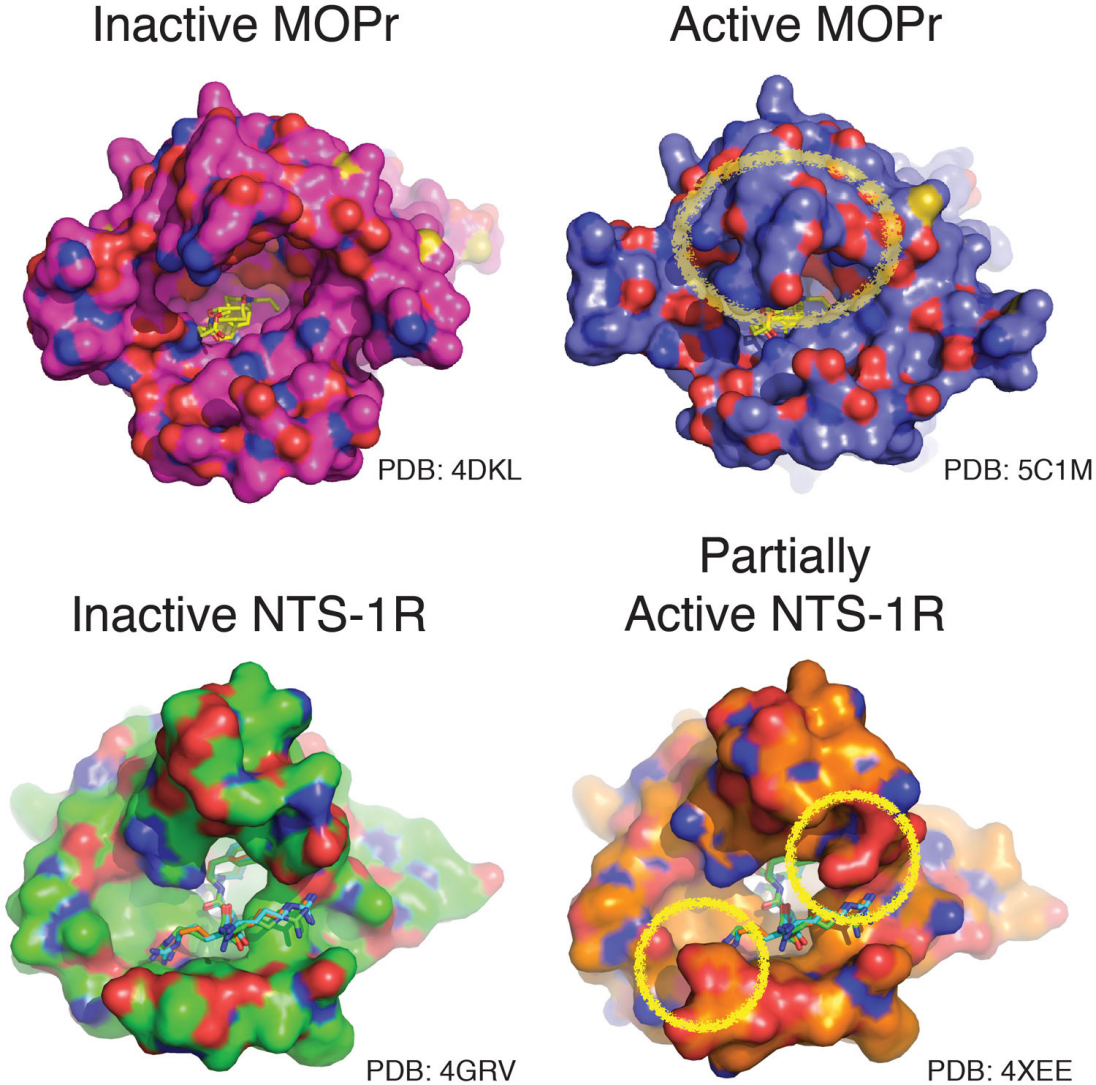
The extracellular regions in the active conformations of peptide hormone/agonist receptors MOPr and NTS-R1 Illustrated are the crystal structures of the inactive and active (or partially active NTS-R1) conformations of the MOPr and NTS-R1 from the top or extracellular view of the receptor. The surface rendering highlights residues or structure on the extracellular face that change upon receptor activation (circled). The mu-opioid receptor (MOPr) in its inactive conformation (purple) is compared to the Nb39-bound (G protein mimic) form in blue. Similarly, the inactive NTS-R1^33^ (green) is compared with a mutant NTS-R1^34^ that adopts a partially active conformation (orange). (MOPr; PDB 4DKL, MOPr-Nb39; PDB 5C1M, NTS-R1; PDB 4GRV and active-like NTS-R1; PDB 4XEE).

**Extended Data Figure 10:**
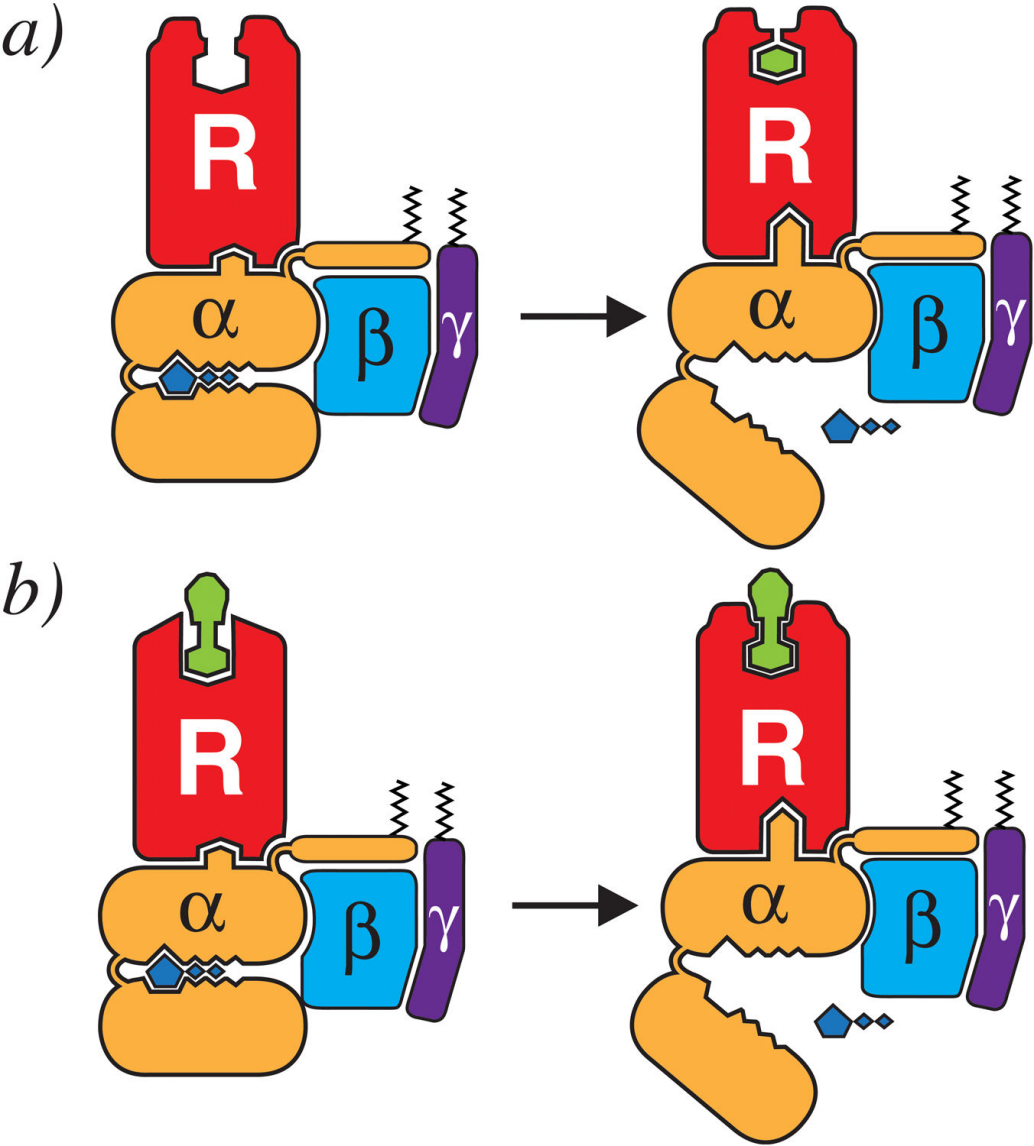
Model of G protein-dependent high-affinity agonist binding As in Figure 5 (of the main text), nucleotide-free G protein-stabilized family A GPCRs experience alterations in the extracellular face of the receptor, thus affecting orthosteric binding site. In a monoamine receptor like the β_2_AR, G protein binding and GDP loss accompanies the stabilization of a closed, active conformation of the receptor, as in ***a)***. ***b)*** For family members such as MOPr or NTS-R1, where the peptide hormones/agonists are considerably larger, the influence of the G protein-mediated changes in the extracellular domain structure result in similar effects on orthosteric ligand dissociation. Rather than closing over the orthosteric site as with monoamine receptors as in ***a)*** the extracellular face may contain structures and residues that ‘pinch’ the larger ligands.

## Supplementary Material

supp_info

supp_videolegends

video1

video2

video3

video4

video5

video6

video7

## Figures and Tables

**Figure 1:**
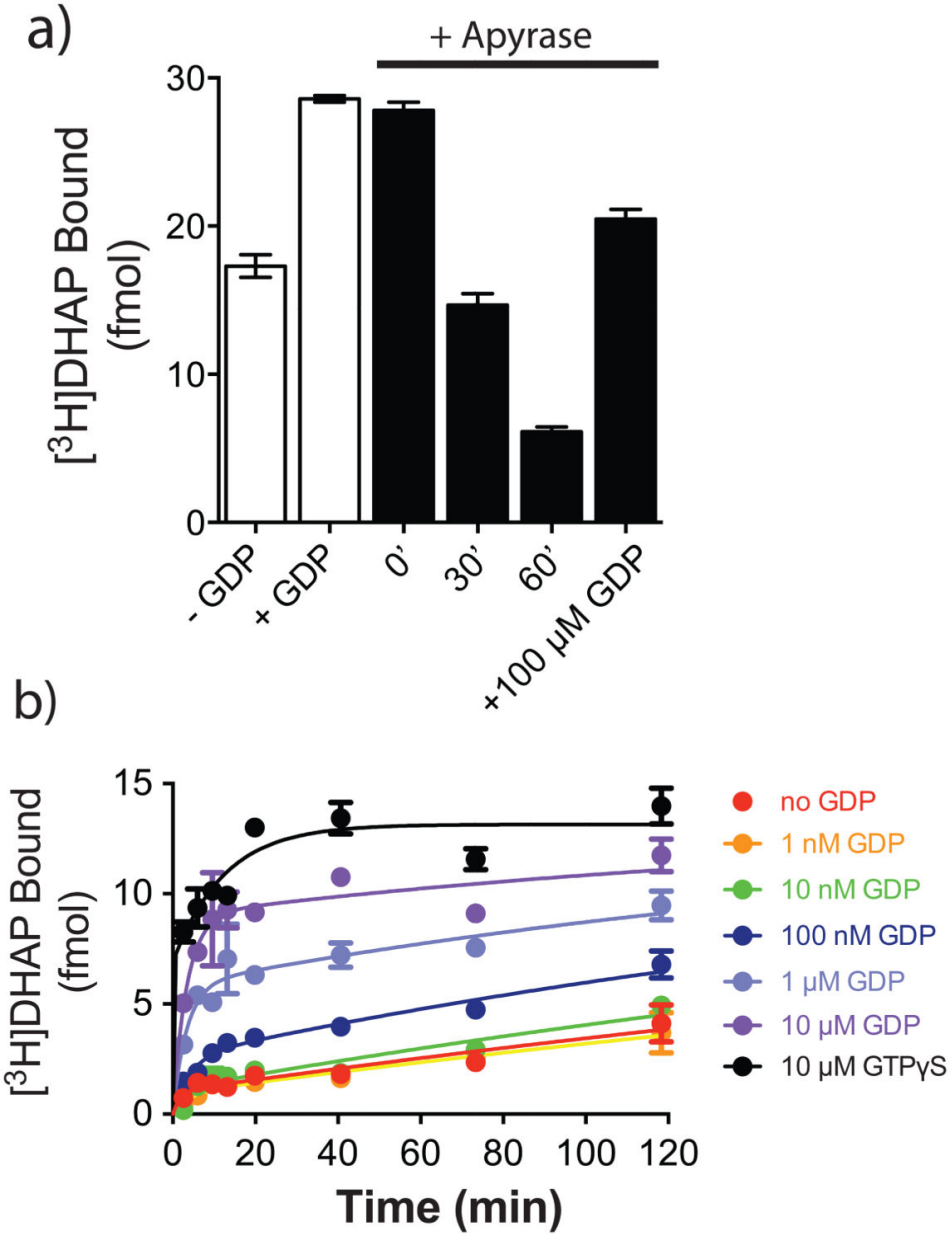
Guanine nucleotides influence antagonist binding to β_2_AR•Gs complexes ***a)*** Binding of 2 nM [^3^H]DHAP to β_2_AR•Gs in the absence or presence of GDP. Addition of apyrase to GDP-bound β_2_AR•Gs led to a progressive decrease in [^3^H]DHAP binding over time, which could be restored with excess GDP. ***b)*** Addition of increasing concentrations of GDP enhances the rate and extent of [^3^H]DHAP binding to apyrase-treated β_2_AR•Gs complexes. Data in ***a)*** are shown as mean ± SEM from n=3 independent experiments performed in duplicate. Data in ***b)*** are representative of three independent experiments.

**Figure 2:**
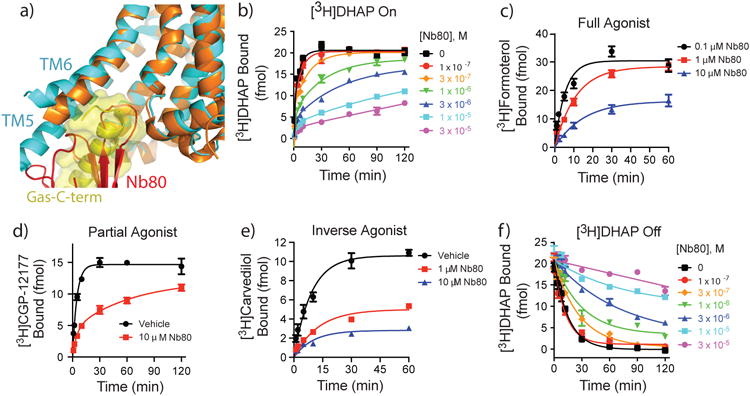
Trapping active-state β_2_AR with Nb80 slows both antagonist and agonist association ***a)*** Nb80 (red) mimics G protein (yellow) in both its binding site and the β_2_AR conformation it stabilizes. The structure of Nb80-bound β_2_AR (3p0g) is shown in orange, Gs-bound β_2_AR (3sn6) in cyan. ***b)*** Pre-incubation of β_2_AR with increasing concentrations of Nb80 progressively slows association of neutral antagonist [^3^H]DHAP to β_2_AR. ***c)*** Nb80 also slows association of full agonist [^3^H]formoterol, ***d)*** partial agonist [^3^H]CGP12177, and ***e)*** inverse agonist [^3^H]carvedilol to β_2_AR. ***f)*** Nb80 stabilizes the closed, active conformation and slows [^3^H]DHAP dissociation from β_2_AR in a concentration-dependent manner. Data in ***b)*** and ***f)*** are representative of three independent experiments. All other data are specific binding, shown as mean ± SEM from n=3 independent experiments performed in duplicate.

**Figure 3:**
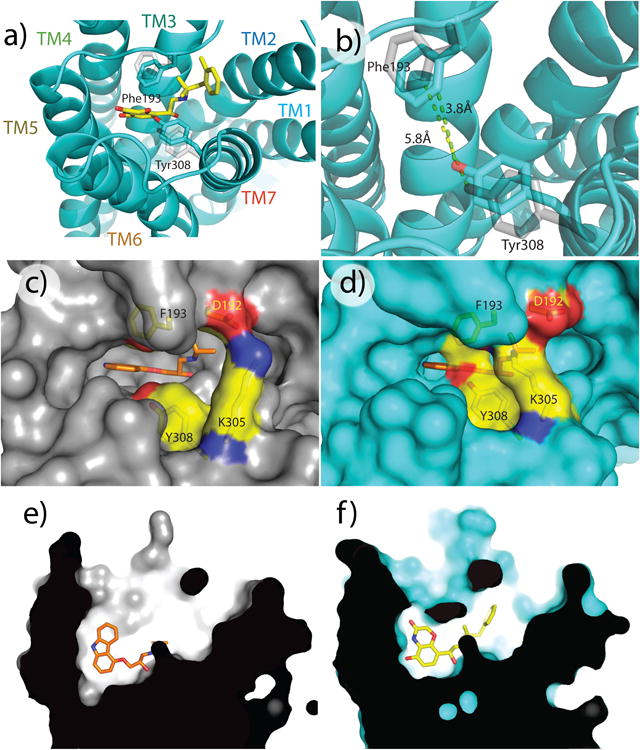
Activation of β_2_AR closes the hormone binding site ***a)*** Stabilization of the β_2_AR active conformation by Gs (or Nb80) brings the side chains of Phe193^ECL2^ and Tyr308^7.35^ closer to one another compared to their positions in structures in the absence of G protein. ***b)*** Closer view of the orthosteric site, highlighting Phe193^ECL2^ and Tyr308^7.35^. Distances (in Ångstroms) between the hydroxyl on Tyr308^7.35^ and 2-carbon on the phenyl ring of Phe193^ECL2^ are indicated. ***c) and d)*** A surface view comparing the extracellular face of β_2_AR in inactive (panel ***c***) or active (panel ***d***) conformations, showing how G protein-stabilized structural rearrangements occlude the hormone binding site in the active state. ***e) and f)*** Cutaway view illustrating closure of the hormone binding site around the bound agonist in the active state. The inverse agonist carazolol is shown in orange, the agonist BI-167107 is shown in yellow.

**Figure 4:**
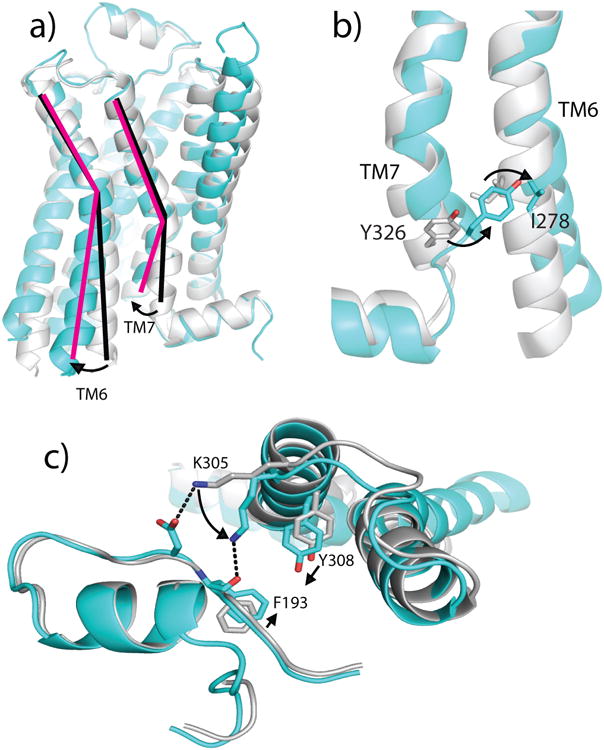
Allosteric communication between β_2_AR G protein- and hormone-binding sites ***a)*** In the β_2_AR active state (cyan), the cytoplasmic end of TM6 moves away from the receptor core by ∼14 Å relative to its position in the inactive-state structure, allowing for an inward movement of TM7. ***b)*** Rotation of TM7 allows Tyr326^7.53^ (of the highly conserved NPxxY motif) to fill the space vacated by the conserved aliphatic residue Ile278^6.40^. ***c)*** The rotation of TM7 repositions Tyr308^7.35^ and Lys305^7.32^. This conformational change allows Lys305^7.32^ to coordinate the backbone carbonyl of Phe193^ECL2^, stabilizing its movement toward Tyr308^7.35^ to form a “lid” over the hormone binding site.

**Figure 5:**
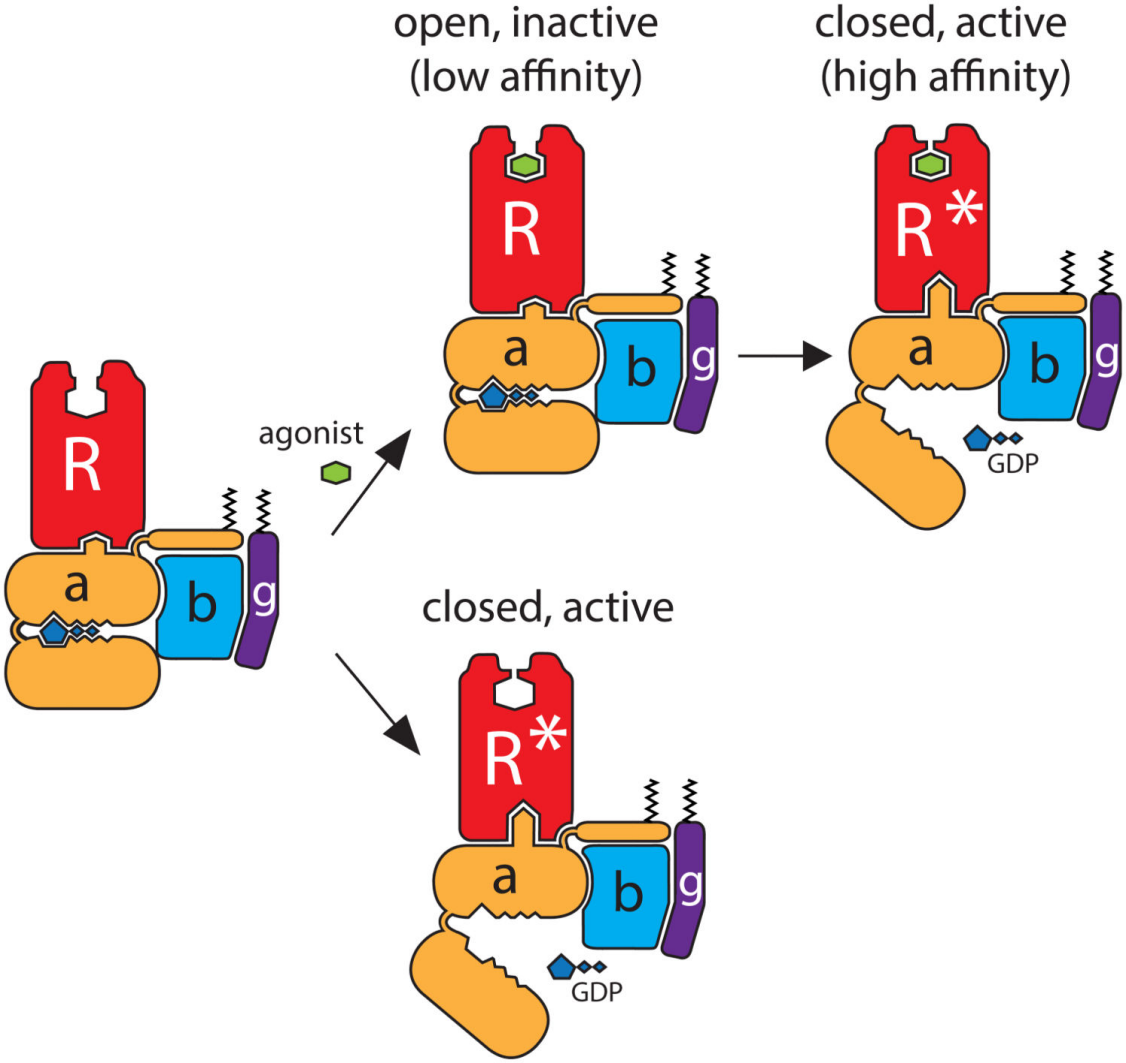
Basis for G protein-dependent high-affinity agonist binding Agonist binding promotes the receptor-G protein interaction and GDP release from Gα. In this nucleotide-free state, the C-terminal helix of Gα remains embedded in the receptor core, stabilizing the conformational changes at both the intracellular and extracellular faces of the receptor. At the extracellular side, the orthosteric binding site closes around the bound agonist, sterically opposing agonist dissociation and thereby enhancing the observed affinity. Constitutive (basal) receptor activity may also activate the G protein, releasing GDP and thereby stabilizing the ‘closed’ conformation of the receptor in the absence of an agonist. See also Extended Data Figure 10.

## Abbreviations

GPCR: G protein-coupled receptor
[^3^H]DHAP: [^3^H]-dihydroalprenolol
GTPγS: guanosine 5′-[γ-thio]triphosphate
