# Supplementary material for: Allosteric coupling from G protein to the agonist binding pocket in GPCRs
Source: Nature. Author manuscript; Available in PMC 2017 Nov 26. (PMC5702553; doi:10.1038/nature18324)
Supplement: supp_info [file NIHMS787243-supplement-supp_info.docx]

**Supplementary Material**

**Supplemental Methods**

*Radioligand Association Experiments using rHDL Particles*

YFP-tagged m-opioid receptor (YMOPr) was purified as described by Kuszak *et al* ^35^. M2 muscarinic receptor (M2R) was purified as described by Haga *et al* ^36^. Reconstitution of purified YMOPr and M2R into rHDL was performed as described above. All assays were performed in Tris-buffered saline (TBS; 25 mM Tris-HCl, pH 7.4, 136 mM NaCl, 2.7 mM KCl) with a final concentration of 0.05% w/v bovine serum albumin. Reaction components were mixed and pre-incubated at room temperature (see below) before the addition of radioligand to initiate the association time course. Aliquots were withdrawn at the indicated times and filtered over Whatman GF/B filters pre-soaked in 0.3% w/v polyethyleneimine. Filters were washed with ice-cold TBS, dried, and subjected to liquid scintillation counting on a TopCount™ NXT (Perkin-Elmer, MA). Bound ligand never exceeded 10% of the total ligand added.

*[^35^S]GTPγS Binding to Apyrase-treated β_2_AR•Gs complexes*

Gs and Flag-tagged β_2_AR were reconstituted in rHDL and treated with the non-specific nucleotide lyase, apyrase. Samples were applied to an anti-Flag affinity resin to remove products of the GDP degradation (GMP and P_i_). Samples were incubated with 100 nM [^35^S]GTPγS at room temperature. At various times, samples were subjected to rapid filtration through glass fiber filters (GF/B) followed by 10 volumes of ice-cold buffer washes containing 10 μGDP. Filters were dried and subjected to liquid scintillation counting (Top-Count™, Perkin-Elmer).

*Binding experiments with [^3^H]DHAP and Gs-β_2_AR nucleotide-free complexes*

For association experiments, gel-filtered samples of apyrase-treated Gs-β_2_AR-rHDL particles were incubated with 5 nM [^3^H]DHAP to bind any receptor that was not complexed with Gs. The experiment was started by adding varying amounts of either GDP or GTPγS. For "equilibrium" binding experiments, samples were incubated with all the indicated components at room temperature for 90 minutes before filtration. Non-specific binding was determined in the presence of 10 μM (+/-)-propranolol.

*Saturation analysis of [^3^H]DHAP binding to Gs-β_2_AR in rHDL treated with apyrase.*

Apyrase-treated Gs-β_2_AR-rHDL particles were subjected to size exclusion chromatography to remove free apyrase and incubated with varying concentrations of [^3^H]DHAP in the absence or presence of 10 μM GTPγS. Samples were incubated for 90 min (at RT) and filtered as above to separate bound from free subjected to liquid scintillation counting.

*[^3^H]N-methylscopolamine Association to M2R•Go*

Purified Go heterotrimer^31^ was added to M2R-rHDL in 20 mM HEPES pH 8.0, 100 mM NaCl, 1 mM EDTA, 1.1 mM MgCl_2_ at an initial ratio of 1:100 M2R:Go and incubated for 30 minutes at room temperature. Go was diluted at least 100-fold to minimize the amount of detergent added. The mixture was then incubated with BioBeads SM2 (BioRad) for 1 hour at 4°C to remove any residual detergent. M2R•Go was diluted in TBS + 1 mM MgCl_2_ and pre-incubated with 10 µM GTPγS or 5 mU/ml apyrase (New England Biolabs) for 1 hour at room temperature before addition of [^3^H]N-methylscopolamine (Perkin Elmer) to 1 nM final concentration. For experiments examining the effect of GDP on [^3^H]N-methylscopolamine binding, GDP was added simultaneously with radioligand to initiate the time course. Non-specific binding was determined in the presence of 10 µM atropine.

*[^3^H]iperoxo Association to M2R*

M2R-rHDL was incubated with the indicated concentrations of Nb9-8 for 30 minutes at room temperature. [^3^H]iperoxo (custom synthesized by Moravek Biochemicals, CA) was added to reach 1 nM final concentration. Non-specific binding was determined in the presence of 10 µM atropine.

*[^3^H]Diprenorphine Association to MOPr•Go*

MOPr•Go in rHDL was prepared as described above for M2R. MOPr•Go was diluted in TBS + 1 mM MgCl_2_ and pre-incubated with 10 µM GTPγS or 5 mU/ml apyrase (New England Biolabs) for 1 hour at room temperature before addition of [^3^H]diprenorphine (Perkin Elmer) to 1 nM final concentration. Non-specific binding was determined in the presence of 10 µM naloxone.

*[^3^H]Diprenorphine Association to MOPr•Nb39*

Purified YFP-MOPr was reconstituted into rHDL as above, and pre-incubated with 100 μM active-state stabilizing nanobody Nb39 for 30 minutes at room temperature. To initiate the time-course, [^3^H]diprenorphine was added to reach 1 nM final concentration. Non-specific binding was determined in the presence of 10 µM naloxone.

*Enrichment of β_2_AR and β_2_AR-Y308A from HEK293T cells*

Frozen membranes were thawed on ice and NaCl, MgCl_2_, and GTPγS were added to reach final concentrations of 300 mM, 1 mM, and 10 µM, respectively. Timolol was then added to a final concentration of 1 µM and the membranes were incubated for 10 minutes on ice. Receptors were solubilized for 1 hour at 4 °C in the presence of 1% dodecylmaltoside (DDM) and 0.1% cholesterol hemi-succinate (CHS). Following centrifugation for 30 minutes at 25,000g, the supernatant was applied to Ni-NTA agarose. The column was slowly washed with 20 column volumes of 20 mM HEPES, pH 8.0, 300 mM NaCl, 0.1% DDM, 0.01% CHS to remove bound timolol. Receptor was eluted in the same buffer plus 200 mM imidazole and concentrated using an Amicon 30 kDa-cutoff spin concentrator for addition to the rHDL reconstitution mixture.

**Supplementary Discussion**

**The Ternary Complex:** Taken together, the data in this report provide an integrated pharmacological and structure-based mechanism for how G proteins stabilize high affinity agonist binding and how agonists are capable of promoting G protein coupling to GPCRs. As predicted by the *simple* ternary complex model, agonist-bound receptors exist in equilibrium between inactive and active states^7-9^. The probability of adopting an active state and, by extension, an agonist’s efficacy to activate G proteins can be described in terms of the allosteric free energy coupling between the agonist- and G protein-binding sites. This energetic linkage is ultimately a representation of conformational changes within the receptor^31^ that are stabilized by the concerted actions of both ligands (agonist and G protein). Indeed, recent biophysical studies suggest that GPCRs are highly dynamic, but the movement of the cytoplasmic end of TM6 to the fully active conformation, a hallmark of GPCR activation, cannot be stabilized by agonists alone, even by saturating concentrations^37-40^. Even a full agonist covalently bound to β_2_AR revealed an inactive receptor conformation by x-ray crystallography, together suggesting that the Agonist-bound active Receptor complex (A•R*) is transient and unstable. Only upon G protein or nanobody coupling (A•R*•G) can populations in the fully active conformation be observed using biophysical and pharmacological approaches.

An alternative hypothesis that perhaps explains why picomolar affinity full agonists and even covalent full agonists fail to stabilize the active conformation may be the existence of sub-states. Here the traditional ternary complex model A•R* may actually be composed of A•R' and A•R* states, where the R* state is rare and unstable. Such activation intermediates are described by Kim *et al.* in which binding of the picomolar affinity full agonist BI-167107 to β_2_AR could not fully stabilize a population of receptors in the active conformation without a G protein or G protein mimetic nanobody^39^. The fully active state was only stabilized in the presence of both agonist and nucleotide-free G protein or G protein mimetic nanobody (A•R*•G). This is also consistent with the fact that most agonist-bound GPCR crystal structures appear in their inactive conformations or display only some aspects of a partially active conformation. The subtle differences likely reflect the difference in the energy landscapes of activation for individual receptors, where certain receptors (e.g. the adenosine A2A receptor^41^) are able to adopt meta-stable activation intermediate states.

The *extended* ternary complex model further postulates that active receptor states, and in turn receptor-G protein interactions, can either be achieved spontaneously (i.e. basal receptor activity) or be stabilized by agonist binding. In support of this prediction, we found that G protein or nanobody binding even in the absence of agonist was sufficient to stabilize a closed, active conformation of the receptor, here demonstrated with a variety of GPCRs. We propose that the closed, active receptor conformation we observe in this case corresponds to the active, coupled states of the receptor (R*•G or A•R*•G) put forth by the ternary complex models. However, neither the *extended* nor the more thorough *cubic* ternary complex models take into account the effect of nucleotides on the receptor-G protein interaction and agonist affinity^20,21^. In this current study we demonstrate that the presence or absence of nucleotide has a profound effect on receptor conformation, particularly in the agonist-free, basally active state.

To this end these data suggest that the extended ternary complex model be further refined to include both the concepts of receptor sub-states of the active conformation (e.g. R' and R*), as well as the role of nucleotides in regulating or stabilizing the A•R*•G complex.

**The closed, active conformation: specific to β_2_AR or applicable to other GPCRs?** The capacity of G proteins to stabilize a closed receptor conformation explains the poorly defined GTPγS-mediated increases in radiolabeled antagonist binding that has been observed with GPCRs of several families, including muscarinic, α-adrenergic, adenosine, and opioid receptors^22–25^ (as in Fig. S7 & S8). We speculate that this observation arises from accumulation of nucleotide-free receptor-G protein complexes formed from basal receptor activity in an environment where free GDP is depleted, such as in isolated plasma membranes. Populations of receptors in their closed, active conformation thus accumulate, thereby limiting accessible antagonist binding sites unless the receptor-G protein complexes are uncoupled with nucleotides (GDP, GTP, or GTPγS). Consistent with this model, purified M2 muscarinic acetylcholine receptor (M2R) stabilized in its active conformation with nucleotide-free Go heterotrimer displays slow association of the antagonist [^3^H]N-methylscopolamine unless GDP or GTPγS are present. Additionally, an active state-stabilizing nanobody, Nb9-8, was able to slow association of the agonist [^3^H]iperoxo to M2R (Fig. S8a & S8b). We also observe slower association rates of [^3^H]diprenorphine to purified mu-opioid receptor (MOPr) in complex with either nucleotide-free Go heterotrimer or a Nb39, a nanobody selective for the active state of MOPr (Fig. S8c). These data support previous observations that MOPr agonist association and dissociation were enhanced by GTP^35,42^, and the finding that the G protein mimetic nanobody Nb39 was able to slow dissociation of the agonist BU72 from MOPr^29^.

The slower binding characteristics are consistent with the recent crystal structure of agonist- and nanobody-bound M2R, which demonstrates a pronounced lid-like structure over the orthosteric site. The lid, composed of three aromatic residues (Y104^3.33^, Y403^6.38^ and Y426^7.39^) forms largely through the inward movements of TM6 and TM7^26^ and can conceivably affect ligand association and dissociation in a manner conceptually similar to what we propose for β_2_AR (see Supplemental Movies SM4 and SM5). In contrast, the crystal structures of peptide receptors such as MOPr or the neurotensin receptor NTS-R1 reveal binding sites that are considerably larger and more open at the extracellular surface than for receptors for small molecules (e.g. monoamines), including β_2_AR^28,33^. However, structures of the nanobody-stabilized MOPr and partially activated (through mutagenesis) NTS-R1 reveal subtle changes in the structure of extracellular face around a peptide ligand that may in essence “pinch” the ligand rather than cap the ligand binding site^29,33,34^. Formation of the active receptor state (Supplemental Movie SM3, SM4, SM5 and Figures S9 & S10) could promote more favorable peptide-receptor interactions that enhance agonist affinity and alter orthosteric ligand association and dissociation.

Thus, despite the subtle structural differences, the influence of G proteins (or nanobodies) on the association and dissociation of ligands at the orthosteric sites are shared, suggesting that the allosteric effects of G proteins on orthosteric agonists may be manifested by conceptually common mechanisms. Interestingly, class A GPCRs with very lipophilic agonists (e.g. rhodopsin, sphingosine-1-phosphate receptor 1 (S1PR1), and free fatty acid receptor 1 (FFAR1)) have a “lid-like” structure over their orthosteric sites even in the inactive state, suggesting that ligand entry occurs from the lipid bilayer^43-45^. Although the mechanism remains unclear, receptors for membrane-partitioned agonists still exhibit nucleotide-dependent shifts in agonist affinity and in some cases accelerated agonist association and dissociation in response to GTP, like the β_2_AR ^46-50^.

Several family B and C GPCRs also show G protein-dependent changes in agonist affinity^51-55^, including recent studies with the metabotropic glutamate receptor mGluR2, where the active conformation of the 7TM domain allosterically stabilizes the active conformation of the extracellular ligand-binding domain^56^. Thus, while the GPCR superfamily exhibits a great deal of structural diversity, reciprocal allosteric communication between the G protein-binding site and the orthosteric site appears to be fundamental to GPCR function. The specific structural mechanisms that allow G proteins to communicate with the orthosteric site of each GPCR will need to be investigated and may differ slightly from the mechanisms described here.

**The Closed Conformation and GPCR-Arrestin Coupling**: One of the hallmarks of GPCR activation, first demonstrated with *meta*II-rhodopsin, is the outward movement of the intracellular regions of the TM domains, most notably TM6. Evidence from EPR spectroscopy^57^ as well as x-ray crystallography support the outward movement of TM6. This outward movement of TM6 in rhodopsin is necessary in order to open a binding cavity capable of accommodating the G protein C-terminus and facilitate GDP release on Gα^58-60^. Similar movements of TM6 were observed in the active conformation structures of the β_2_AR, MOPr and M2R^2,27,29^. The large TM movements on the intracellular face are accompanied by conformational changes on extracellular regions, which for many GPCRs form a ‘lid-like’ structure over the orthosteric site, as described in this current study. Similarly, the recent crystal structures of activated rhodopsin bound to the finger loop peptide of arrestin^61^ and of activated opsin bound to arrestin^62^ reveal similar outward conformational rearrangements of TM6 to accommodate the finger loop region of arrestin. The role of arrestin in stabilizing only the active, chromaphore-bound metaIIb-rhodopsin and not the dark state, nor opsin (retinal-free), suggests an active conformation-specific arrestin interaction^63^. As we have seen with G protein coupling, the conformational changes that accompany arrestin binding could stabilize rearrangements at a receptor's extracellular face to form a lid-like structure over the orthosteric site of Class A GPCRs such as hormone receptors (see Supplemental Move SM6 and SM7). Arrestin has been shown to enhance agonist binding to the β_2_AR and M2R in a manner similar to G proteins, and therefore agonist-receptor-arrestin has been described as an alternative ternary complex^64^. It is thus plausible that arrestin coupling, like G protein coupling, will stabilize a closed receptor conformation and alter agonist dissociation, thereby enhancing agonist affinity for the receptor.

**References**

35. Kuszak, A.J. *et al.* Purification and functional reconstitution of monomeric mu-opioid receptors: allosteric modulation of agonist binding by Gi2. *J. Biol. Chem.* **284,** 26732-41 (2009).

36 Haga, K. *et al.* Structure of the human M2 muscarinic acetylcholine receptor bound to an antagonist. *Nature* **482**, 547-551 (2012).

37. Onaran, H. O., Rajagopal, S. & Costa, T. What is biased efficacy? Defining the relationship between intrinsic efficacy and free energy coupling. *Trends Pharmacol. Sci.* **35,** 639–47 (2014).

38. Nygaard, R. *et al.* The dynamic process of β(2)-adrenergic receptor activation. *Cell* **152,** 532–42 (2013).

39. Kim, T. H. *et al.* The role of ligands on the equilibria between functional states of a G protein-coupled receptor. *J. Am. Chem. Soc.* **135,** 9465–74 (2013).

40. Manglik, A. *et al.* Structural insights into the dynamic process of β2-adrenergic receptor signaling. *Cell* **161,** 1101-11 (2015).

41. Xu, F. *et al.* Structure of an agonist-bound human A2A adenosine receptor. *Science* **332,** 322-327 (2011).

42. Childers, S.R., and Snyder, S.H. Differential regulation by guanine nucleotides of opiate agonist and antagonist receptor interactions. *J. Neurochem.* **34,** 583-93 (1980).

43. Palczewski, K. *et al.* Crystal Structure of Rhodopsin: A G Protein-Coupled Receptor. *Science* **289,** 739-45 (2000).

44. Hanson, M.A. *et al.* Crystal structure of a lipid G protein-coupled receptor. *Science* **335,** 851-55 (2012).

45. Srivastava, A. *et al.* High-resolution structure of the human GPR40 receptor bound to allosteric agonist TAK-875. *Nature* **513,** 124-27 (2014).

46. Bornancin, F., Pfister, C., & Chabre, M. The transitory complex between photoexcited rhodopsin and transducin. Reciprocal interaction between the retinal site in rhodopsin and the nucleotide site in transducin. *Eur J Biochem.* **184,** 687-98 (1989).

47. Devane, W.A. *et al.* Determination and characterization of a cannabinoid receptor in rat brain. *Mol Pharmacol.* **34,** 605-13 (1988).

48. Lefkowitz, R.J. *et al.* Regulation of prostaglandin receptors by prostaglandins and guanine nucleotides in frog erythrocytes. *J. Biol. Chem.* **252,** 5295-5303 (1977).

49. Grandt, R., Aktories, K., & Jakobs, K.H. Guanine nucleotides and monovalent cations increase agonist affinity of prostaglandin E_2_ receptors in hamster adipocytes. *Mol. Pharmacol.* **22,** 320-26 (1982).

50. Sarau, H.M. *et al.* Identification and characterization of leukotriene D_4_ receptors and signal transduction processes in rat basophilic leukemia cells. *J. Biol. Chem.* **262,** 4034-41 (1987).

51. Rodbell, M., Krans H.M., Pohl, S.L., & Birnbaumer, L. The glucagon-sensitive adenyl cyclase system in plasma membranes of rat liver. IV. Effects of guanylnucleotides on binding of 125I-glucagon. *J Biol Chem*. **246,** 1872-6 (1971).

52. Dieterich, K.D., Grigoriadis D.E., & De Souza E.B. Corticotropin-releasing factor receptors in human small cell lung carcinoma cells: radioligand binding, second messenger, and northern blot analysis data. *Endocrinology*. **135,** 1551-8 (1994).

53. Taylor, R.L. & Burt, D.R. Guanine nucleotides modulate TRH-receptor binding in sheep anterior pituitary. *Mol Cell Endocrinol.* **21,** 85-91 (1981).

54. Hill, D.R., Bowery N.G., & Hudson, A.L. Inhibition of GABAB receptor binding by guanyl nucleotides. *J Neurochem*. **42,** 652-7 (1984).

55. Albasanz, J.L., Ros, M., & Martin, M. Characterization of metabotropic glutamate receptors in rat C6 glioma cells. *Eur J Pharmacol*. **326,** 85-91 (1997).

56. Doumazane, E. *et al.* Illuminating the activation mechanisms and allosteric properties of metabotropic glutamate receptors. *Proc Natl Acad Sci USA.* **110,** 1416-25 (2013).

57. Farrens D. L. *et al.* Requirement of rigid-body motion of transmembrane helices for light activation of rhodopsin. *Science* **274,** 768–770 (1996).

58. Scheerer P. *et al.* Crystal structure of opsin in its G protein-interacting conformation. *Nature* **455,** 497–502 (2008).

59. Choe H. W. *et al.* Crystal structure of metarhodopsin II. *Nature* **471,** 651–655 (2011).

60. Standfuss J. *et al.* The structural basis of agonist-induced activation in constitutively active rhodopsin. *Nature* **471,** 656–660 (2011).

61. Szczepek M. *et al.* Crystal structure of a common GPCR-binding interface for G protein and arrestin. *Nat. Commun.* **5,** 4801 (2014).

62. Kang, Y. *et al*. Crystal structure of rhodopsin bound to arrestin by femtosecond X-ray laser. *Nature* **523,** 561-567 (2015).

63. Hofmann, K.P. *et al.* The role of arrestin and retinoids in the regeneration pathway of rhodopsin. *J Biol Chem* **267,** 15701-06 (1992).

64. Gurevich, V.V. *et al.* Agonist-receptor-arrestin, an alternative ternary complex with high agonist affinity. *J Biol Chem.* **272,** 28849-52 (1997).
