# Supplementary material for: Allosteric coupling from G protein to the agonist binding pocket in GPCRs
Source: Nature. Author manuscript; Available in PMC 2017 Nov 26. (PMC5702553; doi:10.1038/nature18324)
Supplement: supp_videolegends [file NIHMS787243-supplement-supp_videolegends.docx]

**Supplementary Video/Movie SM1.** *Activation of the β_2_AR.* Morph of the β_2_AR in its inactive conformation bound to inverse agonist carazolol (PDB: 2RH1) and the β_2_AR in its active conformation bound to agonist BI-167607 and nanobody Nb80 (PDB: 3P0G). For reference, epinephrine is modeled in the orthosteric binding site. Morphs were generated using Chimera (Pettersen *et al*, 2004) and rendered with Pymol (The PyMOL Molecular Graphics System, Schrödinger, LLC). Highlighted are Phe193^ECL2^ and Tyr308^7.35^.

Pettersen, E,F., *et al*. UCSF Chimera--a visualization system for exploratory research and analysis. *J Comput Chem*. 2004 **25**, 1605-12.

**Supplementary Video/Movie SM2.** *Comparison of β_1_AR and β_2_AR.* The closed, active conformation is stabilized by the G protein. Morph of the β_2_AR in its inactive conformation bound to inverse agonist carazolol (cyan, PDB: 2RH1) and the β_2_AR in its active conformation bound to agonist BI-167607 and nanobody Nb80 (PDB: 3P0G). Superimposed on top is a similar morph transitioning between the carazolol-boundβ_1_-adrenergic receptor (β_1_AR, lime green, PDB: 2YCW) and isoproterenol-bound (but not G protein- or Nb-bound, PDB: 2Y03). While the β_2_AR adopts a closed conformation stabilized by agonist and G protein, the β_1_AR bound only to isoproterenol does not. For reference, epinephrine is modeled in the orthosteric binding site. Note that nanobody Nb80 has been omitted from the animation for simplicity. Highlighted are Phe193^ECL2^ and Tyr308^7.35^ on β_2_AR and the conserved residues Phe201^ECL2^ and Phe352^7.35^ on β_1_AR. Morphs were generated using Chimera (Pettersen *et al*, 2004) and rendered with Pymol (The PyMOL Molecular Graphics System, Schrödinger, LLC).

Pettersen, E,F., *et al*. UCSF Chimera--a visualization system for exploratory research and analysis. *J Comput Chem*. 2004 **25**, 1605-12.

**Supplementary Video/Movie SM3.** *Activation of the μ-opioid receptor, MOPr.* The ribbon structure of the mu-opioid receptor (MOPr) in its inactive conformation bound to β-funaltrexamine (cyan, PDB: 4DKL) and the MOPr in its active conformation bound to agonist BU72 and Nb39 (orange, PDB: 5C1M). For reference only BU72 is displayed as spheres. Note that Nb39 is not illustrated for simplicity. Morph between the inactive and active conformations was generated using Chimera (Pettersen *et al*, 2004) and rendered with Pymol (The PyMOL Molecular Graphics System, Schrödinger, LLC).

Pettersen, E,F., *et al*. UCSF Chimera--a visualization system for exploratory research and analysis. *J Comput Chem*. 2004 **25**, 1605-12.

**Supplementary Video/Movie SM4.** *Activation of the M2 muscarinic receptor, M2R.* Top view of the ribbon structure of the M2 muscarinic receptor (M2R) in its inactive conformation bound to antagonist 3-quinuclidinyl benzilate, (QNB, PDB: 3UON) and the M2R in its active conformation bound to agonist iperoxo and nanobody Nb9-8 (PDB: 4MQS). Illustrated are sidechain residues Y104^3.33^, Y403^6.38^ and Y426^7.39^ to highlight the ‘lid-like’ structure over the orthosteric site. Acetylcholine is modeled into the iperoxo binding site and illustrated in stick figure for reference purposes. Note that Nb9-8 is not depicted for simplicity. Morphs were generated between the inactive and active conformations using Chimera (Pettersen *et al*, 2004) and rendered with Pymol (The PyMOL Molecular Graphics System, Schrödinger, LLC).

Pettersen, E,F., *et al*. UCSF Chimera--a visualization system for exploratory research and analysis. *J Comput Chem*. 2004 **25**, 1605-12.

**Supplementary Video/Movie SM5.** *Activation of the M2R side view.* Side view of the ribbon structure of the M2R (above) to highlight the ‘lid-like’ structure over the orthosteric site. Illustrated are side chain residues Y403^6.38^ and Y426^7.39^ moving toward Y104^3.33^ during the formation of the active conformation. Note that TM5 was removed from the rendering so that the tyrosine residues may be easily viewed. Morphs were generated between the inactive and active conformations using Chimera (Pettersen *et al*, 2004) and rendered with Pymol (The PyMOL Molecular Graphics System, Schrödinger, LLC).

Pettersen, E,F., *et al*. UCSF Chimera--a visualization system for exploratory research and analysis. *J Comput Chem*. 2004 **25**, 1605-12.

**Supplementary Video/Movie SM6.** *Stabilization of the active state of rhodopsin by G protein.* Top view of the ribbon structure of bovine rhodopsin in its inactive conformation (PDB:1F88) (Palczewski, K. *et al* 2000) and the photoactivated meta-stable form of rhodopsin bound to the C-terminal fragment of the G protein alpha subunit, transducin (transducin not shown) (PDB:3PAR) (Choe *et al* 2011). Note the pre-existing ‘lid-like’ structure over the orthosteric site formed by the ECL2 and N-terminus. Photoisomerization of 11-cis retinal is illustrated in magenta. Morphs were generated between the inactive and active conformations using Chimera (Pettersen *et al*, 2004) and rendered with Pymol (The PyMOL Molecular Graphics System, Schrödinger, LLC).

Palczewski, K. *et al*. Crystal structure of rhodopsin: A G protein-coupled receptor Science 289: 739-745 (2000).

Choe H. W. *et al.* Crystal structure of metarhodopsin II. *Nature* **471,** 651–655 (2011).

Pettersen, E,F., *et al*. UCSF Chimera--a visualization system for exploratory research and analysis. *J Comput Chem*. 2004 **25**, 1605-12.

**Supplementary Video/Movie SM7.** *Stabilization of the active state of rhodopsin by arrestin.* Top view of the ribbon structure of bovine rhodopsin in its inactive conformation (PDB:1F88) (Palczewski, K. *et al* 2000) and an active mutant of opsin bound to activated arrestin (arrestin not shown) (PDB:4ZWJ) (Kang et al 2015). Note the similarities in conformational changes as with metarhodopsin bound to the C-terminal helix of transducin. Photoisomerization of 11-cis retinal, based on the metarhodopsin structures (Extended data: Movie SM6) has been modeled into the opsin-arrestin structure and is illustrated in magenta. Morphs were generated between inactive and arrestin-bound conformations of rhodopsin using Chimera (Pettersen *et al*, 2004) and rendered with Pymol (The PyMOL Molecular Graphics System, Schrödinger, LLC).

Palczewski, K. *et al*. Crystal structure of rhodopsin: A G protein-coupled receptor Science 289: 739-745 (2000).

Kang, Y. *et al*. Crystal structure of rhodopsin bound to arrestin by femtosecond X-ray laser. *Nature* **523,** 561-567 (2015).

Pettersen, E,F., *et al*. UCSF Chimera--a visualization system for exploratory research and analysis. *J Comput Chem*. 2004 **25**, 1605-12.
